# Recent Update on the Anti-Inflammatory Activities of Propolis

**DOI:** 10.3390/molecules27238473

**Published:** 2022-12-02

**Authors:** Felix Zulhendri, Ronny Lesmana, Steven Tandean, Andreas Christoper, Kavita Chandrasekaran, Ilham Irsyam, Auliya A. Suwantika, Rizky Abdulah, Nasrul Wathoni

**Affiliations:** 1 Center of Excellence in Higher Education for Pharmaceutical Care Innovation, Universitas Padjadjaran, Bandung 45363, Indonesia; 2 Kebun Efi, Kabanjahe 22171, Indonesia; 3 Physiology Division, Department of Biomedical Sciences, Faculty of Medicine, Universitas Padjadjaran, Bandung 45363, Indonesia; 4 Biological Activity Division, Central Laboratory, Universitas Padjadjaran, Bandung 45363, Indonesia; 5 Department of Neurosurgery, Faculty of Medicine, Universitas Sumatera Utara, Medan 20222, Indonesia; 6 Postgraduate Program of Medical Science, Faculty of Medicine, Universitas Padjadjaran, Bandung 45363, Indonesia; 7 Peerzadiguda, Uppal, Hyderabad 500039, Telangana, India; 8 Department of Orthopaedics and Traumatology, Faculty of Medicine, Universitas Sumatera Utara, Medan 20222, Indonesia; 9 Department of Pharmacology and Clinical Pharmacy, Faculty of Pharmacy, Universitas Padjadjaran, Bandung 45363, Indonesia; 10 Department of Pharmaceutics and Pharmaceutical Technology, Faculty of Pharmacy, Universitas Padjadjaran, Sumedang 45363, Indonesia; 11 Research Center of Biopolymers for Drug and Cosmetic Delivery, Bandung 45363, Indonesia

**Keywords:** propolis, bee products, anti-inflammatory, nutraceutical, natural product, phenolics, terpenoids, immune system, inflammation

## Abstract

In recent years, research has demonstrated the efficacy propolis as a potential raw material for pharmaceuticals and nutraceuticals. There is limited report detailing the mechanisms of action of propolis and its bioactive compounds in relation to their anti-inflammatory properties. Thus, the aim of the present review is to examine the latest experimental evidence (2017–2022) regarding the anti-inflammatory properties of propolis. A systematic scoping review methodology was implemented. After applying the exclusion criteria, a total of 166 research publications were identified and retrieved from Scopus, Web of Science, and Pubmed. Several key themes related to the anti-inflammatory properties of propolis were subsequently identified, namely in relation to cancers, oral health, metabolic syndrome, organ toxicity and inflammation, immune system, wound healing, and pathogenic infections. Based on the latest experimental evidence, propolis is demonstrated to possess various mechanisms of action in modulating inflammation towards the regulatory balance and anti-inflammatory environment. In general, we summarize that propolis acts as an anti-inflammatory substance by inhibiting and downregulating TLR4, MyD88, IRAK4, TRIF, NLRP inflammasomes, NF-κB, and their associated pro-inflammatory cytokines such as IL-1β, IL-6, IFN-γ, and TNF-α. Propolis also reduces the migration of immune cells such as macrophages and neutrophils, possibly by downregulating the chemokines CXCL9 and CXCL10.

## 1. Introduction

Propolis is a resinous substance collected by bees to provide physical and biochemical protection to the nests. The biological activities of propolis include antibacterial, antiviral, antifungal, anti-parasitic, antioxidant, and anti-inflammatory. Due to its wide-ranging therapeutic properties for humans, propolis has been used to treat ailments for centuries. Historical records show that ancient Egyptians, Greeks, Persians, Romans, Indians, Mayans, and Australian Aborigines independently developed the therapeutic use of propolis. The more recent scientific research demonstrates that the therapeutic properties of propolis are due to its content of plant secondary metabolites such as phenolics and terpenoids [1,2,3].

The present review article attempts to collate and investigate the recent development of propolis as an anti-inflammatory substance. The term ‘propolis’ in the present study includes propolis from all propolis-producing bees, such as European honeybees (*Apis mellifera*) and stingless bees of the genera such as *Trigona*, *Melipona*, *Geniotrigona*, *Heterotrigona*, and *Tetragonula*.

## 2. Results and Discussion

### 2.1. Immune System Modulation

Inflammation is a coordinated process triggered by the immune system usually in response to microbial infections and/or tissue damage. Inflammatory response is considered successful if the triggers are terminated by an attenuation and resolution of inflammation processes, regulated by anti-inflammatory cytokines and other mediators. However, injurious inflammation occurs if the deleterious inflammatory triggers could not be eliminated and therefore the homeostatic baseline is shifted towards persistent inflammatory environment marked by the constant infiltration of leukocytes and elevated level of pro-inflammatory cytokines [4,5,6]. Propolis with its inflammation-modulating properties could play a role in regulating the components of the immune system (Table 1).

Most of the studies investigating the effect of propolis on the components of the immune system had been carried out in in vitro and ex vivo studies. Propolis appears to have an inflammation-modulating effect on innate immunity. Bueno-Silva [8] demonstrated that in LPS-activated peritoneal macrophages isolated from C57BL6 mice, propolis reduced the expression of inflammatory cytokines such as IL-1α, IL-1β, IL-4, IL-6, IL12p40, IL12p70, IL1-3, monocyte chemoattractant protein-1 (MCP1), and granulocyte-macrophage colony-stimulating factor (GM-CSF) [8]. Interestingly, propolis reduced the expression of several inflammatory genes, such as *Mmp7*, *Egfr*, *Adm*, *Gata3*, *Wnt2b*, *Txn1*, *Herpud1*, *Axin2*, *Car9*, *Id1*, *Vegfa*, *Hes1*, *Hes5*, *Icam1*, *Wnt3a*, *Pcna*, *Wnt5a*, *Tnfsf10*, *Ccl5*, *Il1b*, *Akt1*, *Mapk1*, *Noxa1*, and *Cdkn1b*, while increasing the expression of other inflammatory genes, namely *Cav1*, *Wnt6*, *Calm1*, *Tnf*, *Rb1*, *Socs3*, and *Dab2,* suggesting the complex immune-modulatory properties of propolis that warrant further investigation [8]. 

Several in vivo studies demonstrated the effect of propolis in modulating the immune system towards regulatory profile and anti-inflammatory environment. Propolis stimulated the trans-differentiation of M1 macrophages to D11b^+^, Gr-1^+^ myeloid-derived suppressor cells (MDSCs) in visceral adipose tissue and the peritoneal cavity of lean and obese mice. This trans-differentiation had an anti-inflammatory outcome [29]. In newborn Egyptian-Nubian goat kids, propolis supplementation significantly increased the serum IgG and IgA immunoglobulin levels and reduced the serum pro-inflammatory cytokine levels (IFN-γ, TNF-α, IL-1β, and IL-6) [32]. Hsieh [26] demonstrated that propolis attenuated the increase in neutrophil infiltration caused by intraperitoneal injection of monosodium urate (MSU). Propolis also inhibited the MSU-induced IL-1β, active caspase-1, IL-6, and MCP-1 expression in peritoneal lavage fluid [26].

Moreover, propolis and its bioactive compounds especially artepillin C, increased the TNFR2 expression through the IRF4/cMyc axis in Tregs in mice, suggesting the potential of propolis in up-modulating Tregs for the purpose of attenuating inflammatory diseases induced by auto-immune conditions [30]. In an allergic inflammation mouse model, propolis suppressed the IgE/antigen-induced expression of IL-4, IL-6, and IL-13 in basophils. Phosphorylation of FcεRI signaling molecules Lyn, Akt, and ERK was inhibited in basophils treated with propolis. Propolis also down-regulated IgE mediated- chronic allergic inflammation (IgE-CAI) and attenuated basophils-and-basophil-derived IL-4-induced intestinal anaphylaxis [35].

In addition, propolis maintained the TLR-2, TLR-4, HLA-DR, CD40, and CD80 expression in the monocytes isolated from healthy human subjects. When the monocytes were challenged with MAGE-1 or LPS, propolis inhibited the expression of pro-inflammatory TNF-α and IL-6 and up-regulated the anti-inflammatory IL-10 [12]. Others studies have also confirmed the efficacy of propolis extracts from various geographical locations in reducing the expression of the pro-inflammatory cytokines TNF-α, IL-1β, and IL-6 in macrophage, LPS induced-THP-1,monosodium urate crystals (MSU)-activated THP-1 cells, and LPS-induced human peripheral blood mononuclear cell (PMBC) cell cultures [13,14,16,19,21,22,23,26,28].

Several studies have demonstrated that propolis exhibits several aspects of pro-inflammatory activity in immunosuppressive environment, suggesting the ability of propolis to act as an immunomodulator and/or immune-restoring substance. Oliveira [11] showed that propolis could potentially combat the immunosuppressive effect of chemotherapy drug doxorubicin. Propolis increased the expression of TLR-4, TNF-α, NF-κB, and IL-10 in the monocytes exposed to doxorubicin. Interestingly, the expression of IL-1β and the phosphorylation of IκBα in doxorubicin-exposed monocytes were reduced in the presence of propolis [11]. Further, propolis restored the expression of HLA-DR, TNF-α, and IL-6 in Docetaxel-induced immunosuppression of monocytes [15]. Moreover, propolis was also able to induce the proliferation of lymphocyte, the expression of IL-4 and IFN-γ, and promote antibody response with the dominant IgG1 pattern, comparable to Freund’s and Alum adjuvants in HIV vaccine candidates [34]. 

### 2.2. Cancers

The involvement of inflammatory processes has been well studied and documented in tumorigenesis and carcinogenesis. Inflammation plays a significant role in all stages of cancer development [36,37]. Therefore, the use of anti-inflammatory compounds has been linked to lower the risk of cancers, namely esophageal, stomach, liver, and pancreatic cancer [38]. Propolis, as a natural product, with its anti-inflammatory properties could potentially play a role in alleviating cancer progression and its associated symptoms [3].

In the present review, there are five included studies that are related to role of propolis as an anti-inflammatory substance and cancers. Most are in vitro and in vivo studies. In MDA-MB-231 breast cancer cell lines, propolis has been shown to reduce the expression of Toll-like Receptor-4 (TLR4) signaling pathway molecules, such as TLR4, Myeloid differentiation primary response 88 (MyD88), interleukin-1 receptor-associated kinase 4 (IRAK4), TIR-domain-containing adapter-inducing interferon-β(TRIF), and Nuclear factor kappa B (NF-κB) p65 [39]. Uncontrolled TLR4 activation is linked to the progression in many cancers [40]. Propolis also reduced the expression of pro-inflammatory cytokines tumor necrosis factor-alpha (TNF-*α*), interleukin (IL)-1*β*, and IL-6, and NLR family pyrin domain containing 3 (NLRP3) inflammasome in MDA-MB-231 breast cancer cell lines. In breast cancer animal models, propolis consumption appeared to reduce the population of IL-10 and TGF-β Expressing-CD4+CD25+ Regulatory T cells [41]. The reduction in the expression of IL-10 and TGF- β is thought to be beneficial in cancers as they are both immunosuppressive cytokines that contribute to the development of cancers [42,43].

In addition, propolis down-regulated the expression of NLR family pyrin domain containing-1 (NLRP1) protein and the mRNA of pro-inflammatory cytokines IL-1α,IL-βandIL-18 in human melanoma cell A375 [44]. Moreover, the tumor volume and the multiplicity of colorectal carcinomas in colorectal cancers in rats were reduced by ethanolic extract of propolis. The reduction in tumor volume and multiplicity correlated with the reduction in the expression of the inflammation-associated proteins: inducible nitric oxide synthase (iNOS), TNF-α, NF-κB, and glutathione peroxidase-2 [45]. 

Moreover, Darvishi [46] found that the breast cancer patients that were supplemented with propolis, prior to chemotherapy, had a significant improvement in terms of oxidant/antioxidant balance compared to baseline, based on the measurement of serum IL-2, protein carbonyl, and TNF-α. Conversely, the placebo group patients had significant increase in terms of those pro-inflammatory markers following chemotherapy [46]. Table 2 summarizes the included studies describing the anti-inflammatory activities of propolis in relation to cancers. 

### 2.3. Dental/Oral-Related Disorders and Diseases

Propolis with its antimicrobial, antioxidant, and anti-inflammatory properties has been shown to be efficacious in treating and alleviating dental/oral-related disorders and diseases [48,49]. Peycheva [50] demonstrated that the expression of pro-inflammatory cytokines such as IL-1β, IL-6, TNF-α, IL-17A, IL-18, and INF-γ was significantly reduced in the gingival crevicular fluid isolated from adolescents with moderate gingivitis after receiving toothpaste containing propolis [50]. Similar observations on the effect of propolis in reducing the expression of pro-inflammatory cytokines were also made in studies investigating the effect of propolis on human periodontal ligament cells and human dental pulp cells and tissues [51,52,53]. In addition, propolis appeared to inhibit high glucose-induced NLRP3 inflammasome activation by downregulating the expression of NLRP3, caspase-1 and IL-1β mRNA and their associated protein levels [54]. Moreover, the anti-inflammatory effect of propolis on the expression of pro-inflammatory cytokines such as IL-1β and TNF-αwas also demonstrated in various animal models [55,56]. Table 3 summarizes the use of propolis in dental/oral-related disorders based on its anti-inflammatory properties.

Importantly, human clinical trials were carried out to investigate and demonstrate the use of propolis, especially with regards to its anti-inflammatory properties in dental and oral-related issues.Sukmawati [59] found that in patients subjected to propolis treatment following gingival curettage had better recovery parameters such as improvement in terms of plaque index (PI), probing pocket depth (PPD), bleeding on probing (BOP), and reduction in inflammatory interleukin-1β (IL-1β), when compared to patients treated with the conventional treatment tetracycline [59]. Additionally, in the primary teeth of children aged 5-10 years following pulpotomy, propolis treatment was more efficacious compared to standard treatment Formocresol based on histological examination which showed thick and continuous dentin bridge formation with minimal inflammation induced by leukocyte infiltration [62].

Propolis-containing toothpaste significantly reduced the plaque accumulation and the salivary expression of inflammatory cytokines, especially the IL-1β and IL-6 levels in patients with gingivitis [63]. Propolis was as effective as 0.1% triamcinolone in alleviating pain in erythema in symptomatic oral lichen planus [61]. Kiani [65] demonstrated that propolis mouthwash can effectively decrease gingival inflammation and bleeding, without causing tooth discoloration or staining [65].

Moreover, Lv and Qu (2021) compared the efficacy of propolis and traditional Chinese medicine in alleviating the symptoms of chemotherapy-induced oral mucositis in leukemia patients. They observed that the patients in the propolis treatment had lower incidence of oral mucositis compared to the control group that were given traditional Chinese medicine. In addition, the propolis group patients had significantly shorter oral mucositis recovery time. The mRNA expression of inflammatory cytokines such as IL-22 and TNF-α, and chemokines CXCL9 and CXCL10 were also significantly lower in the propolis group [64]. 

Shabbir [60] investigated the efficacy of propolis paste as an intracanal medicament on postoperative endodontic pain intensities in comparison to calcium hydroxide in patients with single-rooted necrotic teeth with visible periapical. There was no statistically significant difference in terms of the mean pain scores. However, the incidence of flare-up was slightly higher in the propolis group (17% of the patients) compared to calcium hydroxide group (12%) [60].

### 2.4. Metabolic Syndrome-Related Disorders and Diseases

Metabolic syndrome has a set of well-defined criteria as follows: abdominal obesity (population specific), increased triglycerides (≥150 mg/dL or ≥1.7 mmol/L), reduced HDL (<40 mg/dL for men and <50 mg/dL for women), increased blood pressure (systolic ≥ 130 mm Hg and/or diastolic ≥ 85 mm Hg), and increased fasting glucose (>100 mg/dL or 5.5 mmol/L) [66,67,68]. Metabolic syndrome is a precursor of chronic diseases such as type 2 diabetes mellitus, chronic kidney disease, non-alcoholic fatty liver disease, dementia and Alzheimer’s disease, and cardiovascular diseases [69]. The 2017–2018 data showed that metabolic syndrome affects 34% of US population and up to 25% of the population in other developed countries, showing no signs of abating [70]. One of the hallmarks of metabolic syndrome is the chronic inflammation where propolis could potentially play a role in alleviating [69].

The majority of the studies identified and included in the present review that are related to metabolic syndrome were animal models (in vivo) (Table 4). Sakai [71] demonstrated the infiltration of epididymal fat by macrophages and T cells was reduced significantly by propolis consumption in the mice fed with high-fat diet [71]. Propolis was also shown to reduce insulin resistance, body weight gain, and fat accumulation induced by high fat diet in mice. Additionally, propolis significantly reduced the mRNA expression level of pro-inflammatory cytokines TNF-α, IL-1β, and IL-6 in a dose-dependent manner, while increasing the anti-inflammatory IL-10 expression in hepatic and adipose tissues [72]. Similar result was also observed in the hypercholesterolemia rabbits. Propolis consumption reduced the level of C-reactive protein, IL-6, and TNF-α, and inhibited the expression of CD68, TLR4, NF-κB p65, MMP-9, and TNF-α in the carotid arteries [73]. Further, Cardinault [74] found that propolis induced the expression of Nrf2-target genes in adipocytes, transactivated Nrf2 response element and the Nrf2 DNA-binding in mice [74].

Many other animal studies had confirmed the ability of propolis in reducing the level and expression of pro-inflammatory cytokines in metabolic syndrome-related disorders/diseases. However, Cardinault [76] compared three different types of propolis namely poplar, *Baccharis*, and *Dalbergia* and found that poplar propolis reduced the expression of mRNAs coding for TNF-α, chemokine C-C motif ligand 5 (Ccl5), and Ccl2, while *Baccharis* and *Dalbergia* propolis appeared to increase these mRNA expression in high fat-fed mice. Interestingly, the poplar and *Baccharis* propolis had similar polyphenol profile, suggesting it could be other types of bioactive compounds that contribute to this difference in effect [76].

More importantly, several human clinical trials confirm the efficacy of propolis in attenuating inflammation in patients with metabolic syndrome-related chronic diseases. The marker of inflammation MCP-1 (monocyte chemoattractant protein-1) level in urine gradually decreased in the propolis-treated chronic kidney disease patients [85]. Zhu [86] demonstrated that, in a 24-month trial, propolis-treated elderly subjects had significant improvement in Mini-Mental State Exam (MMSE) scores compared to placebo group. The cognitive improvement correlated with the decrease in serum pro-inflammatory cytokines IL-1β, IL-6, and TNF-α. Propolis-treated patients also had an increase in serum TGF-β1 [86]. Zakerkish [88] demonstrated that patients with type 2 diabetes mellitus treated with propolis had significant reduction in the serum level of high-sensitivity CRP (hs-CRP), IL1-β, and TNF-α [88]. Further, Soleimani [89] demonstrated that propolis attenuated hepatic steatosis and fibrosis in non-alcoholic fatty liver disease (NAFLD) patients. Propolis consumption also significantly reduced the serum level hs-CRP in NAFLD patients [89]. Furthermore, Gao [87] found that propolis increased serum glutathione (GSH), flavonoids, and polyphenols, and reduced serum lactate dehydrogenase in diabetic patients. However, serum IL-6 appeared to increase in the propolis group [87].

### 2.5. Organ Toxicity and Inflammation

Inflammatory responses triggered by several factors, namely toxic compounds, pathogens, and damaged cells, can promote acute and/or chronic inflammation in various organs, such as the liver, kidney, lung, brain, cardiovascular, gastrointestinal tract, and reproductive organs, leading to persistent cellular and tissue damage [90,91]. Due to its anti-inflammatory properties, propolis has a potential therapeutic use in this regards (Table 5). Medjeber [92] found that in peripheral blood mononuclear cells isolated from Celiac disease patients, propolis significantly inhibited the expression of nitric oxide and interferon-γ while significantly increasing IL-10 expression. Propolis also downregulated the iNOS expression and the activity of NF-κB and pSTAT-3 transcription factors [92]. In the animal models of ulceritis colitis, propolis had a protective effect in the form of the reduction in inflammatory infiltrates, histological damage, the expression of inflammatory mediators such as TNF-α and NO, and the activity of inflammatory enzymes COX-1 and COX-2 [93,94]. In addition, the inflammatory markers in the colonic inflammation: IL-1β, IL-6, and MCP-1 were reduced by propolis. Propolis also increased the expression of TGF-β. Further, propolis increased the diversity and richness of gut microbiota populations while reducing the population of the unfavorable *Bacteroides* spp. [95]. Propolis also appears to be gastric-protective through similar modes of action [96,97]. 

Moreover, propolis was shown to reduce the level of prostaglandin E2 (PGE2)- a potent inflammatory mediator in the peritoneal exudates and TNF-α in the liver of epirubicin-induced hepatotoxicity in rats [101]. Tsuchiya [106] demonstrated that in acetaminophen-induced hepatocellular necrosis in animal models, propolis significantly reduced inflammatory cell infiltration and down-regulated the mRNA expression of IL-1β and IL-10 [106]. Other groups have also confirmed the hepatoprotective capacity of propolis against hepatotoxic compounds through its anti-inflammatory activities by down-regulating and/or inhibiting the expression of NF-κB, COX-2, transforming growth factor β (TGF-β), Bcl-2, and IL-6 [120,121,125,129,131]. Similar protective mechanisms and anti-inflammatory activities of propolis were also observed in the retina, skin, pancreas, spleen, kidneys, ovary, and heart [100,102,104,108,110,112,118,120,128].

The protective effect of propolis is also observed in lung inflammation caused by environmental toxins. Propolis significantly down-regulated and up-regulated Nrf2 and HO-1 expression, respectively, in a dose-dependent manner in nicotine-induced pulmonary damage [115]. In addition, Barroso [105] investigated the effect of propolis in the chronic obstructive pulmonary disease (COPD) animal models. The mice were exposed to chronic cigarette smokes for 60 days to develop a condition mimicking the chronic obstructive pulmonary disease (COPD) and treated with propolis orally for another 60 days. Histological analyses showed that propolis recovered alveolar spaces, alveolar septa, and elastic fibers. Propolis also increased the expression of MMP-2 while simultaneously decreased the expression MMP-12—inducing tissue repair. Interestingly, propolis promoted the recruitment of leukocytes, including macrophages, withoutROSrelease. Furthermore, the lung repair induced by propolis was demonstrated to be through macrophage polarization (from M1 to M2) and the downregulation of IGF1 expression in a Nrf2-independent manner [105].

The protective effect has been observed in several human clinical trials. Mirsadraee [135] demonstrated propolis consumption significantly attenuated the clinical and physiological symptoms of asthma—often considered as an inflammatory disease. Asthma control test (ACT) score, Forced expiratory volume in 1 s (FEV1), FV1/Forced vital capacity and expiratory flows, and fractional exhaled nitric oxide (FENO) were all significantly improved in the propolis group compared to the placebo. Further, eosinophilia was significantly decreased in the propolis patients whereas the placebo group recorded a significant increase during the trial period [135]. 

Further, Silveira [137] showed that propolis supplementation significantly reduced inflammatory marker hs-CRP level in hemodialysis patients. In addition, safety parameters such as amylase, aspartate aminotransferase (AST) and creatine phosphokinase (CPK) were not affected [137]. However, Matsumoto [136] found that propolis supplementation in the elderly women suffering from rheumatoid arthritis did not improve disease severity in terms of Disease Activity Score in 28 joints using erythrocyte sedimentation rate (DAS28-ESR). Moreover, propolis also did not improve any of the secondary endpoints such as DAS28 using C-reactive protein, simplified disease activity index, clinical disease activity index, ultrasonographic evaluation of synovitis, activities of daily living, quality of life, changes in cytokine levels, and adverse events [136]. 

### 2.6. Pathogenic Infections

In the light of evolution, inflammation is a conserved process and an important first line of defense in invertebrates as well as vertebrates against pathogenic invaders. Therefore, inflammation affects both the host and the pathogens, and an uncontrolled inflammation has been shown to be one of the primary drivers of many diseases [138,139]. Reboucas-Silva [140] demonstrated that propolis reduced the viability of *Leishmania (Viannia) braziliensis* promastigotes and parasite burden inside the infected macrophages. Dry propolis extract significantly modified the inflammatory profile of the macrophages by downregulating the TGF-β and upregulating TNF-*α* levels, whereas the alcoholic and glycolic extracts upregulated the IL-10 level [140]. This suggests different types of propolis extracts modulate inflammatory signaling pathway differently (Table 6). 

In addition, Dos Santos Thomazelli [141] investigated the effect of propolis on Human-derived peripheral blood mononuclear cells from American Tegumentar Leishmaniasis (ATL) patients and healthy donors. They found that propolis pre-treatment increased the expression of IL-4 and IL-17 and downregulated IL-10, either in the presence or absence of the *L. braziliensis* infection [141]. Moreover, propolis reduced the expression of IL-1β and NLRC4 inflammasome in the bone marrow-derived macrophages through autophagy following *Pseudomonas aeruginosa* [143]. Propolis was also demonstrated to reduce *Helicobacter pylori* infection-induced increase in pro-inflammatory interleukins and mediators, namely IL-8, IL-12, IL-1β, TNF-α, COX-2, and iNOS. Propolis also inhibited the phosphorylation of ERK, JNK, and p38 MAPKs in *H. pylori*-infected cells in a dose-dependent manner [144]. 

Similar effect was also observed in many animal studies, including cystic echinococcosis, *Escherichia coli* infection, bovine herpesvirus-1(BHV-1) infection, and anthrax. Propolis reduced the expression of pro-inflammatory mediators and cytokines such as iNOS, TNF-α, and NF-κB/p50, IL-2 and IFN-γ [145,146,147,149]. AlGabbani [148] demonstrated that in mice infected with *Plasmodium chabaudi*, propolis inhibited oxidative stress by downregulating the malondialdehyde (MDA) and upregulating the catalase (CAT) activity and the glutathione (GSH) levels. However, propolis appeared to increase the level of pro-inflammatory cytokines such as IFN-γ, TNF-α, granulocyte-macrophage colony-stimulating factor (GM-CSF), and granulocyte colony stimulating factor (G-CSF), suggesting different pathogens and different types of propolis extracts could play a role in shifting inflammatory balance [148].

### 2.7. Wound Healing

Propolis with its anti-inflammatory properties could play a significant role in wound healing process [49]. Most of the studies that were identified and included in this particular theme are in vivo studies (Table 7). Corrêa [150] found that wounded animals that were given propolis had significant improvement in wound healing. The animals in the propolis group had less neutrophil and macrophage in the wounded tissue. In addition, they also had reduced expression of inflammatory transcription factor pNF-κB protein, and inflammatory cytokines, such as TGF-β, TNF-α and IL-6 [150]. Additionally, Sahib [151] showed that propolis-treated wound had significantly increased epithelial closure rate accompanied by decreased level of neutrophils and macrophages and increased level of fibroblasts and blood vessels, compared to the non-treated group [151]. 

Conversely, there were studies demonstrating that propolis induced inflammation during the wound healing process followed by the substantial decrease in inflammation. It appears that the up-regulation of inflammation results in speedier wound healing progression. Marquele-Oliveira [152] found that propolis-enriched cellulose membrane induced higher level of inflammation (neutrophil infiltration) in the wound compared to control group animals in the second day. However, the propolis group had substantially less inflammation in the seventh day and had faster rate of wound healing [152]. In addition, Picolotto [153] observed increased level of inflammation in the form of the upregulation of TNF-α and TGF-β and the recruitment of leukocytes at day 7 in the propolis-treated animal model of diabetic wounds. These inflammatory markers became statistically insignificant compared to control group at day 14. More importantly, the wound healing and closure were significantly better in the propolis-enriched cellulose membrane group [153].

Additionally, Eyarefe [154] found that propolis treatment appeared to induce higher level of inflammatory response in the wound. Propolis induced higher level of inflammatory infiltrates such as monocytes, macrophages, eosinophils, mast cells, and platelets. However, this inflammatory response regressed at day 8 and induced significantly better wound healing compared to the untreated control group. The control group had significantly worse wound healing response and persistent elevation of inflammation through to day 16 [154]. Moreover, the effect of solvents and/or carriers of the propolis extract should also be considered. Saritaş [155] found that the effect of propolis on bowel wound healing animal models with regards to the pro-inflammatory cytokines was less clear due to the solvent effect [155]. 

In terms of human clinical trials, Mujica [156] demonstrated the efficacy of propolis-containing spray in treating human diabetic foot wounds. Propolis-treated patients had significantly higher reduction in wound area: ~4 cm^2^, compared to control group: ~ 3 cm^2^; approximately 33.3% higher reduction in wound area in propolis treated patients over the course of the trial (8 weeks). In addition, propolis also improved the inflammatory profile of the tissues. Propolis treatment significantly increased the glutathione (GSH) and GSH/glutathione disulfide (GSSG) ratio, reduced the pro-inflammatory TNF-*α*, and upregulated the anti-inflammatory IL-10 levels of the treated tissue [156].

**Table 7:** Anti-inflammatory properties of propolis in wound healing.

Geographical Sources of Propolis	Types of Extract	Bioactive Compounds	Measured Outcome	References
**In vivo**				
Brazil	Hydroethanolic extract	Phenolic acids, phenolic terpenes and flavonoids (especially catechins, flavonols, chalcones, isoflavones, isoflavans, pterocarpans and bioflavonoids	Wound healing properties. The animals were wounded and fed 100 mg propolis extract/kg body weight of propolis daily for 9 days. Significant improvement in wound healing following propolis administration, measured in terms of skin area. The animals in the propolis group also had less neutrophil and macrophage infiltration in the wounded tissues. The propolis group also had reduced expression of inflammatory transcription factor pNF-κB protein, and inflammatory cytokines, such as TGF-β, TNF-α and IL-6.	[150]
Brazil	Ethanolic extract	*p*-coumaric acid and artepillin C	In wound healing models, there was a severe inflammatory infiltration in all groups, with the BC/PP membrane group showing the most. When compared to the other groups, the BC/PP samples revealed higher levels of neutrophil involvement on the second day. The greater level in the BC/PP group exhibited a substantial decrease in neutrophil infiltration by the seventh day. Propolis-enriched membrane induced an intense pro-inflammatory action when compared to BC membrane and SHAM in the beginning of the treatment, followed by a significant reduction in the inflammatory process during the follow-up of the wounds. Propolis induced a faster wound healing rate.	[152]
Brazil	Ethyl acetate and butane extract	Benzoic acid, anisaldehyde, daidzein, liquiritigenin, isoliquiritigenin, formononetin, medicarpin, vestitol, isovestitol, neovestitol, biochanin A, vesticarpan, retusapurpurin	TNF-α levels in red propolis-stimulated wounds were considerably greater at day 7. Expression of pro-inflammatory cytokines and TGF-β (anti-inflammatory) peaked 7 days after injury, and pro-inflammatory cytokine and TGF-β expression were greater in the groups treated with bacterial cellulose membrane combined with red propolis. Propolis also promoted leukocyte recruitment. These inflammatory responses induced faster wound healing.	[153]
Brazil	Ethanolic extract	Not determined	Tissues exposed to cements containing EERP had a tissue reaction similar to that of the tissues exposed to cements without propolis, demonstrating that they were biocompatible in rat subcutaneous tissue. The addition of propolis resulted in healing, and propolis did not interfere in the repair process.	[157]
Brazil	Not determined	Not determined	Propolis did not affect the wound healing process in terms of the presence of inflammatory cells, fibroblasts, collagen, neovascularization, granulation tissue and re-epithelialization.	[158]
Indonesia	Propolis gel	Not determined	The topical application of propolis gel upregulated the expression of FGF-2 and fibroblasts in promoting ulcer healing in streptozotocin-induced diabetic rats.	[159]
Iran	Ethanolic extract	Not determined	Oral propolis alone and in combination with 30 ppm SNPs provided anti-inflammatory effects and increase fibroblast proliferation and collagen deposition in experimental wounds thus increasing the rate of healing. Propolis + 60 ppm SNPs had a cytotoxic effect.	[160]
Iran	Ethanolic extract	Flavonoids, phenols, terpenoids, steroids, alkaloids, resins, saponins, coumarins	A significant decrease was observed in the numbers of neutrophils and macrophages while a significant increase was observed in number of fibroblasts and blood vessels. Epithelial closure rate was significantly increased in the EEP-treated wounds. Powerful effect of propolis on accelerating wound healing was observed as the healing process of excision wound in albino rats treated with the EEP ointment started earlier and had a faster course than the comparative group.	[151]
Iran	Oral propolis (100mg/kg)	Not determined	The rate of wound healing decreased significantly in the propolis group by day 12. By day 12, there was a significant decrease in number of neutrophil and eosinophil. By day 12, there was a significant increase in fibroblast count and collagen fiber density.	[160]
Nigeria	Not determined	Flavonoids, anthraquinones, cardiac glycosides, steroids, terpenoids, and alkaloids	After the rats were anesthetized, each rat’s dorsum was cleaned with alcohol and chlorhexidine before being prepared for aseptic surgery. For each treatment, three identical circular full-thickness skin incisions measuring 6.5 ± 0.5 mm in diameter were made on the dorsum of each rat. According to the experimental plan, two drops (0.1 mL) of each agent (propolis, propylene glycol, silver sulfadiazine) were administered to the wounds twice a day. The inflammatory cellular response was much greater (*p* = 0.002) in the propolis extract group. Monocytes and macrophages were significantly higher in the PE group (*p* = 0.003). Other inflammatory cells such as eosinophils, mast cells, and platelets were much more prevalent in the PE group (*p* = 0.001). However, the inflammatory infiltration regressed at day 8 in the propolis treated group, whereas the untreated group had signs of inflammation through to day 16. The propolis treated group had the highest rate of wound healing.	[154]
Nigeria	Hydroethanolic extract	Not determined	In MRSA-infected wound model rats, propolis promoted wound healing and bacterial clearance with regression of neutrophil infiltration and attenuated platelet reduction.	[161]
Portugal	Ethanolic extract	Not determined	Wound scratch assay using NHDF cells presented better results with Propolis Extract 2 at 0.5%. The samples also promoted cell migration, demonstrating the wound-healing potential. A significant reduction in the margins of the scratch was observed, maximum with Propolis Extract 2 at 0.5%.	[162]
Turkey	Ethanolic extract	Not determined	In bowel wound healing animal models, propolis appeared to have beneficial effect on wound healing. However, the effect of propolis on inflammatory cytokines such as TNF-α, IL-1, and IL-6 was less certain, perhaps due to the presence of ethanol solvent in the propolis preparation.	[155]
Turkey (Eğriçayır^®^)	Water-Based Propolis Extract	Not determined	In rabbits, the treatment of Amniotic membrane transplantation + Topical water-based propolis extract shows significant improvement in the defect area of the corneal alkali burn eyes. The wound healing process of corneal alkali burn eyes was shown to be sped up by the combination of propolis and amniotic membrane. There was no difference in the severity of the inflammatory alterations between the Amnion membrane transplantation + Propolis group, the Propolis only therapy group, and the control group.	[163]
**Human clinical trial**				
Chile	Not determined (propolis spray)	Not determined	From the examination of diabetic wound tissues applied with propolis spray, TNF-α was significantly decreased and IL-10 was increased. Propolis promoted a reduction in the wound’s area by an average of 4 cm^2^ compared to control patients. The healing was related to an increase in the connective tissue deposit.	[156]

### 2.8. Others

The anti-inflammatory properties of propolis have also been demonstrated in wide-ranging studies (Table 8). In vitro studies demonstrated the inhibition of propolis extract from various geographical sources in terms of the hyaluronidase activity [164]. In addition, propolis extracts also downregulated the expression of pro-inflammatory cytokines, such as TNF-α, IL-6, IL-1β, MCP-1, ICAM-1, and VCAM-1, and their associated gene expression, including miR-19a-3p, miR-203a-3p, and miR-27a-3p [165]. Further, an in vivo study found that propolis reduced the expression of *H1R* and IL-9 genes, protein kinase Cδ (PKCδ), and nuclear factor of activated T-cells (NFAT) signaling pathways in rhinitis animal model [166]. In another allergic animal model, propolis reduced IL-13 production and eosinophilic infiltration and induced the frequency and number ofpolymorphonucler myeloid-derived suppressor cells (PMN-MDSC) [167]. 

In terms of human clinical trials, Fudhali [134] demonstrated that propolis compress significantly reduced phlebitis incidence associated withintravenous therapy [134]. Susan [168] reported the potential use of propolis as an anti-nociceptive therapy post-surgery [168]. Moreover, Soleimani [169] found that propolis supplementation reduced the pro-inflammatory cytokine IL-6 level in male military cadets subjected to Cooper 12-min run test and running-based anaerobic sprint test [169].

**Table 8:** Anti-inflammatory properties of propolis in other types of conditions.

Geographical Sources of Propolis	Types of Extract	Bioactive Compounds	Measured Outcome	References
**In vitro and ex vivo**				
Brazil/*Melipona orbignyi*	Hydroethanolic extract	*O*-coumaroyl *O*-galloyl-hexoside, *O*-coumaroyl *O*-galloyl-hexoside, Aromadendrin, Di-*O*-galloyl *O*-coumaroyl-hexoside, *O*-cinnamoyl *O*-galloyl-hexoside, Di-*O*-galloyl *O*-cinnamoyl-hexoside, Di-*O*-coumaroyl-hexoside, Naringenin, Di-*O*-coumaroyl *O*-galloyl-hexoside, Methyl aromadendrin, *O*-Cinnamoyl-*O*-coumaroyl-hexoside, *O*-Cinnamoyl *O*-coumaroyl *O*-Galloyl-hexoside, Methyl naringenin, terpenoids	Inhibition of hyaluronidase activity	[164]
Brazil/ *Melipona quadrifasciataanthidioides*	Hydroethanolic extract	Coumaroyl-galloyl-hexoside, Coumaroyl-galloyl-hexoside, Aromadendrin, Digalloyl-coumaroyl-hexoside, Cinnamoyl-galloyl-hexoside, Digalloyl-cinnamoyl-hexoside, Dicoumaroyl-hexoside, Naringenin, Dicoumaroyl-galloyl-hexoside, Methyl aromadendrin, Cinnamoyl-coumaroyl-hexoside, Cinnamoyl-coumaroyl-galloyl-hexoside, terpenoids	Inhibition of hyaluronidase activity	[170]
Brazil	Ethanolic extract	Caffeic acid, *p*-coumaric acid, ferulic acid, dihydrokaempferol, dicaffeoylquinic acid, pinobanksin, pinocembrin, kaempferol, drupanin, pinobanksin-3-*O*-acetate, chrysin, quercetin, luteolin, artepillin C, cinnamic acid derivatives, apigenin, galangin	Measuring the effect of brown and green propolis on the expression of miRNAs associated with inflammatory responses. 1. miR-19a-3p and miR-203a-3p, which target mRNA coding for TNF-α, were upregulated by propolis. The increase in both miRs reduces the expression of TNF-α. 2. miR-27a-3p which regulates NFE2L2 expression, was increased by propolis.	[165]
Brazil	Ethanolic extract (green propolis)	*p*-Coumaric acid, Dicaffeoylquinic acid isomer, Dicaffeoylquinic acid, Quercetin, Pinobanksin, 4′-methoxy Pinobanksin, Kaempferol, Quercetin-methyl ether, 5-isoprenyl caffeic acid, 3-isoprenyl-*p*-Coumaric acid, 3-hydroxy-2,2-dimethy-8-prenylchromane-6-propenoic, Caffeic acid isoprenyl ester, Pinocembrin, 3-hydroxy-2,2-dimethy-8-preylchromane-6-propenoic isomer, Galangin, Kaempferide, Pinobanksinr-3-*O*-acetate, Quercetin-dimethyl ether, 5-isoprenyl caffeic acid-*p*-coumaric acid ester, 3-hydroxy-2,2-dimethy-8-preylchromane-6-propenoic isomer, Diisoprenyl -*p*-Coumaric acid isomer, Artepillin C (3,5-diisopentenyl-4-hydroxycinnamic acid), 3-Prenyl-4-(dihydrocinnamoyloxy)-cinnamic acid, (E)-3-[2,3-dihydro-2-(1-methylethenyl)-7-prenyl-5-benzofuranyl]-2-propenoic acid, triterpenes(4 types)	Artepillin C was the most abundant component (relative content 35.68%). The survival rates of the cells were significantly increased when the EEP-B was at the concentrations of 5, 10 and 20 μg/mL, and the survival rate was the highest at 20 μg/mL. After EEP-B treatment, the levels of IL-6 and TNF-α decreased significantly. At a concentration of 20 μg/mL EEP-B, the expression levels of these cytokines were the lowest.In the EEP-B treatment group, the levels of MCP-1, ICAM-1 and VCAM-1 also decreased significantly, especially at a concentration of 20 μg/mL.	[171]
Brazil, Spain, Poland, China, Portuguese, Slovania, Morocco, Greece, Ethiopia, Thailand. Hungary, Italy, South America, Tunisia, Japan	Ethanolic extract	Flavones, flavonols, Flavanones, dihydroflavonols, gallic acid, caffeic acid. Catechin, clorogenic acid, *p*-coumaric acid, ferulic acid, naringenin, quercetin, apigenin, kaempferol, pinocembrin, CAPE, Galangin	Hyaluronidase inhibitory activity ranged from 0% to 68.20% with a propolis concentration of 10 mg/mL. ACE inhibitory activity was higher than 95% for all the samples except for one propolis (78%), which contained the lowest amount of flavanols (27.89 mg C/g)	[172]
Cameroon, Central Africa	Dichloromethane methanol extract	2-Hydroxy-8-prenylbiochanin A, 2′,3′-Dihydroxypropyltetraeicosanoate, Triacontyl *p*-coumarate, β-amyrine, Oleanolic acid, β-amyrine acetate, Lupeol, Betulinic acid, Lupeol acetate, Lupeol acetate	β-amyrine acetate and lupeol acetate were found to be the potent inhibitor of ROS with an IC_50_ values (4.3 ± 0.3 and 1.1 ± 0.1 µg/mL), respectively, compared to the Ibuprofen (11.2 ± 1.9 µg/mL) as a standard anti-inflammatory drug. Lupeol acetate was found to be the most potent inhibitor of ROS. The new compound, 2-hydroxy-8-prenyl biochanin A, was found to be a potent inhibitor of nitric oxide (IC_50_ = 23.3 ± 0.3 µg/mL).	[173]
Egypt	Ethanolic extract of propolis	1-(2,4-dihydroxy-6- methoxyphenyl)-3-phenylprop-2-en-1-one (22.83%); hexadecanoic acid (14.75%); hexadecanoic acid, ethyl ester (6.50%); 1,2-benzenedicarboxylic acid, diisooctyl ester (3.96); 2,4,6-triphenyl-1,3-dioxane (3.29%); 1,1,4,7- tetramethyldecahydro-1H-cyclopropa[e]azulen-4-ol (2.89%); octadecanoic acid, ethyl ester (2.13%); cyclopropaneoctanoic acid, 2-[(2-pentylcyclopropyl)methyl]-, methyl ester, trans,trans- (1.88%); dodecanoic acid, 4-penten-1-yl ester (1.87%); 9-octadecenoic acid, (2-phenyl-1,3-dioxolan-yl) methyl ester, cis (1.77%); 2- naphthalenemethanol,1,2,3,4,4a,5,6,7-octahydro-à,à,4 a,8- tetramethyl-, (2R-cis)- (1.69%); 2-propenoic acid, 3-(4-hydroxy-3-methoxyphenyl) (1.65%); Ar-turmerone (1.59%); 3-phenyl-2-propene-1-ol (1.54%); 1H-purine-2,6-dione, 3,7- dihydro-3,7-dimethyl- (1.52%); 3-hydroxy-4- methoxycinnamic acid (1.35%); 5-azulenmethanol, 1,2,3,4,5,6,7,8-octahydro-à,à,3,8-tetramethyl- (1.31%); á-sitosterol (1.28%); pentadecanoic acid, 14-methyl-, methyl ester (1.19%); 1,4-dimethyl-7-propan-2-ylidene-2,3,4,5,6,8- hexahydro-1H-azulene (1.17%); 1-butyl-2-methyl-7- methoxy-5h,6h-pyrido[3,4-b]indol (1.14%); dasycarpidan-1- methanol, acetate (ester) (1.13%).	From the assessment of the inhibition of the activity of histamine release, the concentration of 1000 µg/mL promoted the greatest value of anti-inflammatory activity (71.14%), while the concentration of 7.81 µg/mL promoted the lower potential (12.93) of anti-inflammatory activity with an IC_50_ of 68.52 µg/mL.	[174]
Japan	Ethanolic extract	Nymphaeol-A, nymphaeol-B, nymphaeol-C, isonymphaeol-B, and 3′-geranyl-naringenin	The bioactive compounds inhibited the albumin denaturation, nitrite accumulation, and cyclooxygenase-2 (COX-2) and α-glucosidase activity. Nymphaeol-A and isonymphaeol-B inhibited acetylcholinesterase activity.	[175]
Lithuania	Ethanolic extracts	Not determined	In primary rat cerebellar neuronal-glial cell cultures affected by ischemia, propolis significantly protected the cultures from hypoxia-induced elevation of TNF-α, IL-1β and IL-6.	[176]
Mexico	hexane, ethyl acetate, methanol	Pinostrobin, izalpinin, cinnamic acid, pinocembrin, kaempherol, 3,3-dimethylallyl caffeate in mixture with isopent-3-enyl caffeate, 3,4-dimethoxycinnamic acid, rhamnetin, and caffeic acid	Anti-inflammatory activity.	[177]
**Animal models**				
Brazil	Ethanolic extract	Artepillin C, baccharin, culifolin	In allergic rhinitis animal model, propolis reduced the expression of *H1R* and IL-9 genes. Propolis suppressed protein kinase Cδ (PKCδ) and nuclear factor of activated T-cells (NFAT) signaling pathways.	[166]
Brazil	Hydroethanolic extract	Total phenolics	Propolis treatment reduced peritoneal adhesion and the expression of TNF-α, IL-1β, IL-6, TGF-β1, VEGF, NO and MDA, while increasing the GSH levels compared with the vehicle group.	[178]
Brazil	Hydroethanolic extract	Coumaric Acid, Rutin (quercetin-3-O-rutinoside), Pinobanksin, Quercetin, Kaempferol, Apigenina, Pinocembrin, Pinobanksin-3- acetate, Chrysin, Galangin, Techtochrysin, Artepillin C^®^ and Baccharin (Benzenepropanoic acid, 4-[(E)-2-carboxyethenyl]-2-(3-methyl-2-buten-1-yl) phenyl ester)	In both the low protein diet + propolis group and the standard protein diet + propolis group, green propolis was able to sustain a low inflammatory infiltrate within 7 days, but it was unable to do so within 15 days in the organisms with protein deficiencies.	[179]
Brazil China	Ethanolic and methanolic extracts	Chlorogenic acid, caffeic acid, isochlorogenic acid A, isochlorogenic acid C, myricetin, quercetin, kaempferol, apigenin, pinocembrin, caffeic acid phenethyl ester, galangin, artepillin C.	In LPS-induced mice, propolis reduced the expression of pro-inflammatory cytokines TNF-α, IL-1β, and IL-6.	[180]
Brazil	Hydro alcoholic extraction	Caffeic acid (1.90 ± 0.014 mg/g), p-coumaric acid (10.016 ± 0.028 mg/g), 3,5-dicafeoyl quinic (3,5-DCQ) (14.293± 0.081), 4,5-DCQ (18.364± 0.164), aromadendrin-4-Omethyl-ether (2.519± 0.023), drupanin (17.343± 0.072), artepillin C (50.299± 1.039) and baccharin (8.459± 0.281)	Propolis treatment (OVA+Propolis) reduced the total cell number into the BALF compared to allergic group (OVA) and decreased the number and the frequency of eosinophils in the BALF. Propolis treatment also induced a decrease in IL-13 production on lymph node cell culture re-stimulated with OVA compared to non-treated allergic mice. A significant decrease in the mucus production as well as in the cellular infiltration were found in propolis-treated allergic mice. Together these show anti-inflammatory role of propolis in the OVA-induced Th2 inflammation.This reduction was followed by a decrease in the frequency and number of eosinophils. propolis treatment induced a significant increase in frequency and number of PMN-MDSC.	[167]
Brazil	Hydroethanolic extract	Not determined	In intra-abdominal adhesion rat models, propolis, delivered as intraperitoneal meshes, did not have any effect on adhesions and histological characteristics including inflammatory characteristics.	[181]
Brazil	Hydroethanolic extract volatiles	Hydroethanolic extract: caffeic acid, coumaric acid, naringenin, garbanzol, isosakuranetin, artepillin C, baccharin Volatiles: monoterpenes and sesquiterpenes, such as nerolidol (12.51%), spathulenol (8.64%), cineole (7.42%), β-caryophyllene (5.52%), β-bourbunene (5.14%), γbisabolene (5.11%), α-copaene (4.81%), terpineol (4.78%), acetophenone (4.57%), and α-chamigrene	Both hydroethanolic extract and volatiles derived from propolis had analgesic and anti-inflammatory properties measured using formalin test and carrageenan-induced mechanical hypernociception. The effect was shown to be related to the effect of propolis on the central nervous system.	[182]
China	ethanolic extract	Not determined	The cytokine-cytokine receptor interaction pathway was significantly altered only in female mice, with four annotated genes being down-regulated (CCL21a, CCL21b, CCL21c, and TNFRSF25) and five being up-regulated (HGF, IL-1R1, IL6RA, CNTFR, and EDA2R) in the group treated with ethanol and co-administered with propolis.	[183]
Indonesia	Hydroethanolic extract	Not determined	In skin graft rat models, propolis reduced the serum IL-6 levels.	[184]
Not determined	Not determined	Not determined	Propolis reduced inflammation markers in LPS-induced rats.	[185]
**Human clinical trials**				
Indonesia	Propolis compress	Not determines	The propolis compress treatment group had the highest percentage with a score of 0 or no phlebitis (60%) and the lowest with early signs of phlebitis (40%).	[134]
Indonesia (*Geniotrigonathoracica*)	Hydroglyceric extract	Not determined	Case report. Anti-nociceptive effect post-surgery.	[168]
Iran	Ethanolic extract	180 mg polyphenols and 134 mg flavonoids	In male military cadets subjected to Cooper 12-min run test and running-based anaerobic sprint test, propolis reduced the pro-inflammatory cytokine IL-6 and the IL-6/IL-10 ratio (compared to placebo group).	[169]

## 3. Method

The search strategy for the present review was carried out systematically. Three independent authors (F.Z., A.C., and K.C.) performed the searches for peer-reviewed manuscripts published from 2017 up to May 2022. The guiding statement was as follows: Propolis as an anti-inflammatory substance. The databases searched were Scopus, Pubmed, and Web of Science.The keywords used were: (Propolis or bee glue) AND (inflammat* or inflammas* or cytokine or chemokine). We excluded the terms that describe the individual bioactive compounds of propolis such ascaffeic acid phenethyl ester (CAPE), pinocembrin, and quercetin, as we opted to focus on the experimental evidence using propolis as a whole. Moreover, the exclusion criteria were review articles, in silico articles, non-English language documents, research papers that describe the combination of propolis with other compounds and/or ingredients. 

**Figure 1:**
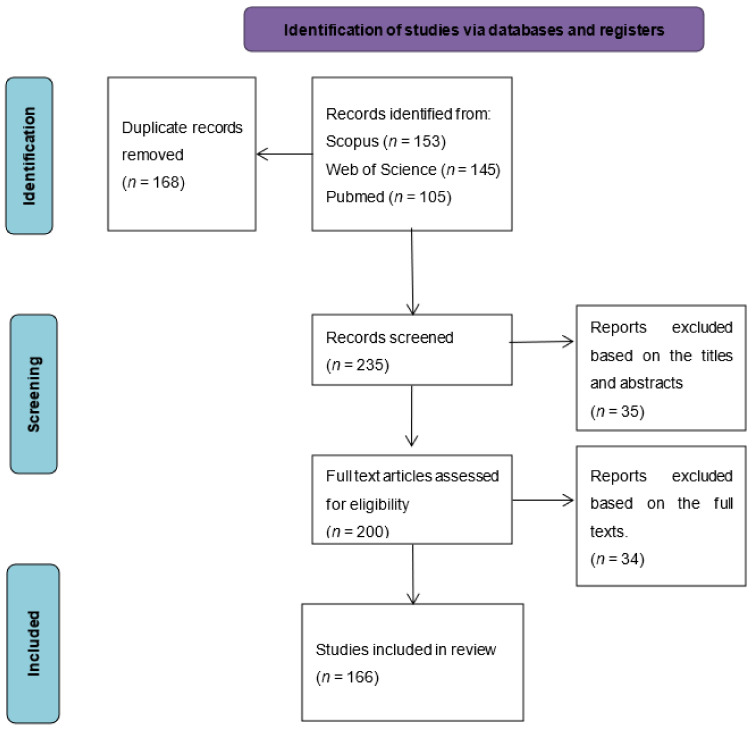
The flow chart of the identification and selection process.

All articles describing the potential use of propolis as an anti-inflammatory substance; in vitro, ex vivo, in vivo, and human clinical trials were selected. The studies were recorded in Mendeley and the duplicates subsequently removed. The selected articles were then screened by analyzing the titles, keywords, abstracts, and full texts. The articles that did not fit in the guiding statement and the set criteria were then removed. If any disagreement arose on the eligibility of a particular article, the disagreement was resolved through the discussion with the other authors. The following data were then collected and tabulated in Microsoft Excel: types of propolis extract, bioactive compounds, geographic locations of the propolis source, types of study, outcome of the study, and references. The authors (F.Z., A.C., and K.C.) then categorized the included studies into the appropriate themes: cancers, dental/oral-related disorders and diseases, immune system modulation, metabolic syndrome-related disorders and diseases, organ toxicity and inflammation, pathogenic infections, wound healing, and others. These categories were formed based on the apparent general themes illustrated by the included studies. Figure 1 illustrates the search and selection processes. 

## 4. General Discussion and Conclusions

The inflammatory signaling cascades and processes are initiated by the disruption of tissue homeostasis, usually by pathogenic infection or tissue damage. The damage signals such as pathogen-associated molecular patterns (PAMPs) and damage-associated molecular patterns (DAMPs) are then recognized by TLRs. TLRs subsequently activates MyD88-dependent signaling pathway by inducing the phosphorylation of the inhibitory IκB protein by IKK. Consequently, NF-κB is activated by its release from IκB protein and translocated into the nucleus. NF-κB then upregulates the expression of the inflammation-related genes. As a result, various cytokines and chemokines, that are intimately involved in inflammation modulation, such as IL-1β, IL-6, IL-8, IL-10, IL-12, IL-15, IFN-γ, and TNF-α are produced. These cytokines then promote the migration of neutrophils and monocytes to the site of injury/infection. In addition, mast cells and macrophages induce further migration by releasing leukotrienes, histamines, and prostaglandins. Moreover, neutrophils also release non-specific toxic compounds such as reactive oxygen and nitrogen species (ROS/RNS) and proteases that are detrimental towards the pathogens and the host cells and tissues. Macrophages and dendritic cells then phagocytose the antigens. These cells subsequently migrate to lymphoid tissues and induce the differentiation of naïve T cells (Th0) into various effector and regulatory cells, such as Th1, Th2, Tregs, and Th17 cells, which in turn induce the production of other sets of cytokines. Finally, the resolution of inflammation is initiated when the neutrophil-induced switch of leukotrienes (produced by macrophages and other immune cells) to lipoxins. In addition, the expression of Fas ligand, protectins, and resolvins promotes the apoptosis and neutrophils. Apoptotic neturophils and other cellular debris are then phagocytosed by macrophages [186,187,188].

**Figure 2:**
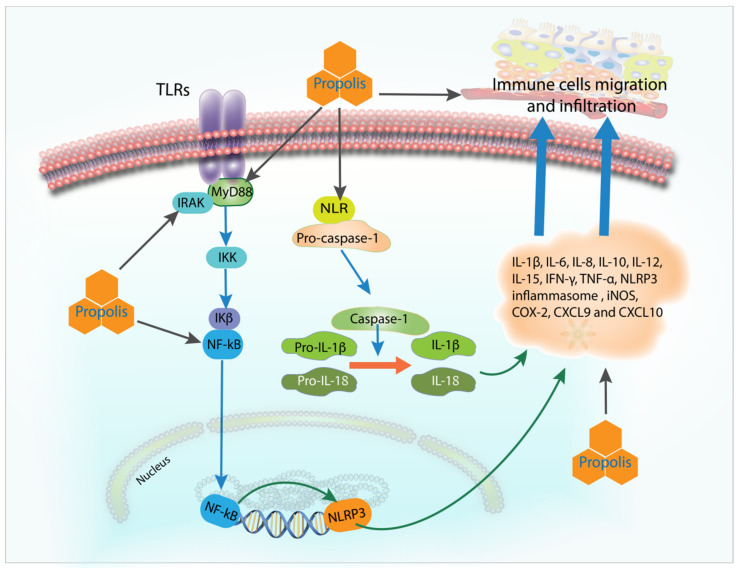
Various mechanisms of action of propolis in modulating inflammation towards the regulatory balance. This figure is constructed based on the biochemical and molecular markers investigated in the 166 included papers. Propolis appears to inhibit and downregulate TLR4, MyD88, IRAK4, TRIF, and NLRP inflammasomes, and their associated pro-inflammatory cytokines such as IL-1β, IL-6, IFN-γ, and TNF-α. Propolis also decreases the migration of immune cells such as macrophages and neutrophils, possibly by downregulating chemokines CXCL9 and CXCL10.

Inflammation involves many complex signaling pathways that are tightly regulated and therefore an uncontrolled, unresolved, and chronic inflammation could be significantly detrimental to the host. Figure 2 (summarized from Table 1, Table 2, Table 3, Table 4, Table 5, Table 6, Table 7 and Table 8) demonstrates various mechanisms of action of propolis in modulating inflammation towards the regulatory balance, anti-inflammatory environment, and consequently, speedier resolution. In general, propolis acts as an anti-inflammatory substance by inhibiting and downregulating TLR4, MyD88, IRAK4, TRIF, NLRP inflammasomes, and their associated pro-inflammatory cytokines, such as IL-1β, IL-6, IFN-γ, and TNF-α. Propolis also reduces the migration of immune cells, such as macrophages and neutrophils, possibly by downregulating chemokines CXCL9 and CXCL10.

An opposite response is observed in some of the studies related to wound healing. Propolis appears to promote an intense inflammatory response in the initial wound healing process. However, the inflammation is then markedly reduced shortly after the initial burst. As a result, propolis-treated wounds are generally healed significantly faster compared to untreated wounds. More importantly, the anti-inflammatory (and immune-modulating) properties of propolis have been shown, not only in in vitro, ex vivo, and in vivo studies, but also in various human clinical trials with consistent results such as the reduction in serum and tissue inflammatory markers: IL-1β, IL-6, TNF-α, and hs-CRP, and the reduction in immune cell infiltration in the inflammation sites. Moreover, the anti-inflammatory effect was demonstrated in the studies using propolis extracts sourced from a wide-ranging of geographical sources and types of bees, solidifying the consistency of the anti-inflammatory properties of propolis.

In the present review, the authors adopted a comprehensive and systematic search strategy in order to objectively fulfill the aim of the study. A broad range of studies from all relevant fields of science and technology was collected and analyzed. The authors limited the search to studies that were published from 2017–2022, to provide coverage of the latest experimental studies in the field. However, the authors only assessed and included English language articles, which could potentially lead to missing studies from non-English databases, as it is clear that most studies originated from non-English speaking countries. The reviewers also did not assess the quality of the included studies in order to include as many studies and to provide as broad coverage as possible. Moreover, the reviewers did not perform any meta-analysis due to the heterogeneity of the included studies.

The authors also noted that a proportion of the included studies did not identify the types of extract, geographical locations, and/or bioactive compounds of the propolis extracts used in the studies (Table 1, Table 2, Table 3, Table 4, Table 5, Table 6, Table 7 and Table 8). We consistently come across this trend in the field of propolis research. In order to solidify the development and application of propolis, including reproducibility, as nutraceutical and/or pharmaceutical products, we suggest that all propolis research should include at least three types of identification, namely bee species, geographical sources, and types of extract. If possible, chemical analyses such as total phenolics and/or flavonoids should be included.

## Figures and Tables

**Table 1:** Anti-inflammatory properties of propolis in terms ofimmune system modulation.

Geographical Sources of Propolis	Types of Extract	Bioactive Compounds	Measured Outcome	References
**In vitro and ex vivo**				
Australia/*Tetragonulacarbonaria*	Methanolic extract	Gallic Acid, *O*-methyl-aromadendrin, dihydroxydihydroflavone, flavones	Propolis inhibited the ionomycin-stimulated production of leukotriene LTB_4_ from human neutrophils.	[7]
Brazil	Hydroethanolic extract	Formononetin, vestitol, neovestitol, biochanin A, quercetin, liquiritigenin, isoliquiritigenin and daidzein	In LPS-activated peritoneal macrophages, obtained from C57BL6 mice, propolis reduced NO production by 65% without affecting the cell viability and decreased the production IL-1α, IL-1β, IL-4, IL-6, IL12p40, IL12p70, IL1-3, MCP1 and GM-CSF. Propolis reduced the expression of *Mmp7*, *Egfr*, *Adm*, *Gata3*, *Wnt2b*, *Txn1*, *Herpud1*, *Axin2*, *Car9*, *Id1*, *Vegfa*, *Hes1*, *Hes5*, *Icam1*, *Wnt3a*, *Pcna*, *Wnt5a*, *Tnfsf10*, *Ccl5*, *Il1b*, *Akt1*, *Mapk1*, *Noxa1* and *Cdkn1b* and increased the expressionof *Cav1*, *Wnt6*, *Calm1*, *Tnf*, *Rb1*, *Socs3* and *Dab2*.	[8]
Brazil	Not applicable	Neovestitol	In macrophages, neovestitol at 0.22 mM inhibited NO production by 60% and the expression of GM-CSF, IFN-c, IL-1b, IL-4, TNF-α and IL-6 levels and increased IL-10 production. These cytokines profile changes were associated with the downregulation of transcription of genes involved in nitric oxide production, NF-κB, IL-1β, and TNF-α signaling pathways: *Nox1*, *Ifnb1*, *Capsns1*, *IL1b*, *Egr1*, *Calm1*, *Elk1*, *Chuk*, *Tnfsf13b*, *Cd70*, *Il19*, *Fos*, *Il18*, *Csf2*, *Il1f6*, *Cd86*, *Tnfsf13*, *Tnfsf18*, and *Cd80* NF-κB and MAPK signaling pathways inhibition and decreased levels of TIRAP were also observed.	[9]
Brazil	Propolis extracted with gum arabic	2′-hydroxy-4′,7-dimethoxy-isoflavane; 2′,7-dihydroxy-4′-methoxy-isoflavane; 2′,4′-dihydroxy-7-methoxy-isoflavane; 4′,7-dihydroxy-2′-methoxy-isoflavane; 2′,4′,4-trihydroxy-chalcone, and lup-20(29) -en-3-ol	Propolis downregulated inflammatory angiogenesis and modulated inflammatory cells by reducing mastocytes and increasing macrophages.	[10]
Brazil (*Melipona fasciculata* Smith)	Ethanol extract	Not determined	Geopropolis treatment and its combination with doxorubicin significantly increased TLR-4 expression in monocytes. Doxorubicin and geopropolis alone significantly increased TNF-α and IL-10 production in human monocytes in the presence of LPS. In contrast to the stimuli alone, the combination of doxorubicin and geopropolis increased the production of TNF-α and IL-10. Geopropolis reduced IL-1β production in the absence of LPS; however, IL-1β production increased in treated monocytes when treated with doxorubicin while remaining at baseline levels when combining with geopropolis. In the presence or absence of LPS, all stimuli reduced the synthesis of IL-6. In comparison to the control, cells treated with doxorubicin, geopropolis, or both raised the expression of the NF-κB gene, and geopropolis reduced the phosphorylation of IκBα.	[11]
Brazil	Hydroethanolic extract	Not determined	In monocytes isolated from healthy subjects, propolis maintained the TLR-2, TLR-4, HLA-DR, CD40 and CD80 expression in the monocytes. When the monocytes were challenged with MAGE-1 or LPS, propolis inhibited the expression of TNF-α and IL-6 and upregulated IL-10. Propolis also down regulated the expression of LC3 induced by LPS.	[12]
Brazil	Hydroethanolic extract	Artepillin C	Propolis extract reduced TNF-α level in LPS-stimulated macrophage culture.	[13]
Brazil	Hydroethanolic extract	Not determined	In LPS-induced THP-1 cell cultures, propolis upregulated a HMOX-1 expression but did not affect pro-IL-1*β* expression. Propolis inhibited CD86 expression stimulated by DNFB.	[14]
Brazil	Hydroethanolic extract	Caffeic acid, dihydrocinnamic acid, benzoic acid, flavonoids, triterpenes, prenyl-*p*-coumaric acid, artepillin C	In monocyte cell cultures, propolis attenuated the immunosuppressive effect of Docetaxel by restoring the expression of HLA-DR, TNF-α, and IL-6.	[15]
Cameroon, Ghana, Indonesia, United Kingdom	Ethanolic Extract	Not determined	Propolis extract reduced the pro-inflammatory cytokines production of TNF-α, IL-1β, and IL-6 in LPS-activated THP-1-derived macrophage cells TNF-α production was considerably reduced with P-UK1, P-C, and P-Ind2, while propolis extracts P-UK4 and P-C had the best anti-IL-1β effects. All propolis extracts strongly reduced the production of the pro-inflammatory IL-6.	[16]
China	Hydroethanolic extract	Vanillic acid, *p*-coumaric acid, trans-isoferulic acid, 3,4-dimethoxycinnamic acid, morin, quercetin, luteolin, galangin, CAPE, apigenin	Propolis downregulated the expression of pro-inflammatory genes of inflammatory cytokines, TNF-α and IL-8.	[17]
Indonesia (*Tetragonula* sp.)	Ethanolic extract	Deoxypodophyllotoxin, Kurarinone, 6-Dehydrogingerdione, 6-Epiangustifolin, Adhyperforin, alpha-Tocopherol Succinate, Xanthoxyletin.	Soft propolis microcapsule (SPM): 50, 100, and 200 mg/kg rough propolis microcapsule (RPM): 25, 50, and 100 mg/kg. For both forms of propolis microencapsulation (SPM and RPM), a substantial reduction in paw volume was seen, indicating a potential anti-inflammatory activity of the SPM and RPM. Due to the significance of inflammatory inhibition, SPM produced a stronger anti-inflammatory activity than RPM.	[18]
Indonesia (*Tetragonula sapiens*)	Ethanolic extract	Not determined	In LPS-induced RAW 264.7 macrophages cells, propolis reduced the production of tumor necrosis factor-alpha (TNF-α), inducible nitric oxide synthase (iNOS), and nitric oxide (NO)	[19]
Iran	Ethanolic extract	Caffeic acid, galangin, quercetin, chrysin	The expression of Cox2 was significantly inhibited by co-treatment with PEEP on RAW 264.7 cells in all tested dosages (*p* < 0.001). In considerable doses of PEEP (0.15 and 1.5 µg/mL), treatment with PEEP resulted in an inhibitory effect on the production of IL-1β. PEEP therapy at a dosage of 15 µg/mL substantially reduced the expression of IL-6 mRNA.	[20]
Italy	Hydroethanolic extract	Galangin, pinocembrin, CAPE	IL-1β and IL-6 levels in human peripheral blood mononuclear cells (PMBC) were slightly increased by PP at a concentration of 25 µg/mL. In LPS-induced human PMBC, PP at 25 µg/mL significantly decreased IL-1β, and IL-6, and markedly downregulated TNF-α; at a dose of 5 µg/mL, PP significantly decreased TNF-α. CAPE (25 µg/mL) decreased IL-1β, IL-6, and TNF-α levels.	[21]
Korea	Ethanolic extract	Not determined	Propolis reduced LPS-induced NO and PGE2 production in a dose-dependent manner (20 µg/mL) in RAW 264.7 macrophages.	[22]
Malaysia (*Heterotrigonaitama*)	Ethanolic extracts	Terpenoids	In RAW 264.7 macraphoages, the *H. Itama* propolis terpenoid-rich extract showed relative low antioxidant effect but inhibited inflammatory response by decreasing the inflammatory mediators iNOS, IL-1β, IL-10, and increasing the antioxidant mediators HO-1. The study also revealed strong anti-inflammatory activity of terpenoids extracted from the propolis sample.	[23]
Morocco	Hydroethanolic extracts	All samples: Caffeic acid, *p*-coumaric acid, pinobanksin, pinocembrin, chrysin,pinobanksin-3-*O*-acetate Specific compound: MP1 (from Boulemane): pinobanksin-5-methyl-ether (3, *m/z* 285) MP2 (from Sefrou): apigenin, caffeic acid pentyl ester, pinobanksin-3-*O*-propionate, pinobanksin-3-*O*-butyrate, and pinobanksin-3-*O*-pentanoate MP3 (from Moulay Yaâcoub): benzoyl hydroxyphenyl acetic acid MP4 (from ImmouzzerMermoucha): sterubin, 3-prenyl-*p*-coumaric acid, dihydrokampferide, capillartimisin A, and isosakuranetin	All of the propolis extracts under investigation demonstrated anti-inflammatory activity in the murine macrophage (RAW 264.7) cell line, with IC_50_ values ranging between 14 and 52 µg/mL in the inhibition of NO production. Sample MP2, which has a greater concentration of bioactive substances such as phenolic acid derivatives and flavones, and sample MP3, which has an IC_50_ value of 29 µg/mL, both exhibit the greatest levels of activity.	[24]
Morocco	Not determined	Caffeic acid, p-coumaric acid, ferulic acid, naringenin, pinocembrin, chrysin, galangin, pinobanksin, quercetin	TNF-α and IL-6 release in LPS-stimulated human peripheral blood mononuclear cells was totally suppressed by PNM treatment, reaching control levels at extract concentrations of 250 µg/mL, while the production of IL-10 was enhanced by 15-fold at the same concentration of propolis.	[25]
Taiwan	Ethanolic extract	Not determined	In a dose-dependent manner (10µg/mL), TGP reduced IL-1β secretion from Monosodium Urate Crystals (MSU)-activated human THP-1 macrophages, mouse J774A.1 macrophages, and mouse bone marrow-derived macrophages (BMDM). In the culture medium of THP-1 macrophages, TGP decreased the expression levels of IL-18, IL-1β, and active caspase-1 (p10). In MSU-activated THP-1 macrophages, TGP decreased the expression levels of IL-6 and monocyte chemoattractant protein (MCP1). In BMDM J774A.1 macrophages, TGP dramatically reduced IL-1β secretion triggered by ATP, nigericin, MSU, and Cholesterol Crystals (CC). In J774A.1 macrophages triggered by ATP, nigericin, MSU, and CC, TGP also decreased the expression levels of active caspase-1 (p10). TGP suppresses the NLRP3 inflammasome as well as the release of IL-1β from J774A.1 macrophages and BMDM in response to intracellular poly(dA/dT), FLAST (flagellin from Salmonella typhimurium), MDP, and LPS. TGP inhibits proIL-1β expression that is produced by LPS in a dose-dependent manner. TGP demonstrated dose-dependent inhibition of LPS-induced TNF-α and IL-6.	[26]
Turkey	Hydroethanolic extract	Pinocembrin, galangin, CAPE, naringenin, caffeic acid, kaempferol, chrysin, rhamnentin, quercetin, 3-*O*-methylquercetin, apigenin	In RAW 264.7 macrophage cells, propolis inhibited NO production in nitrite assays.	[27]
United Kingdom (UK)	Ethanolic extracts	Not determined	The propolis extracts suppressed the secretion of IL-1β and IL-6 with less effect on TNFα. Propolis also reduced the levels of nitric oxide formed by LPS-stimulated macrophages. Propolis extracts exert an anti-inflammatory effect by lowering the production of IL-10, inhibition of pro-inflammatory cytokines and by the metabolic reprogramming of LPS activity in macrophages.	[28]
**In vivo**				
Brazil	Ethanolic extract	Artepillin C, baccharin, kaempferide, drupanin, *p*-coumaric acid, culifolin, CAPE, chlorogenic acid, kaempferol, pinocembrin, narigenin, chrysin	Propolis stimulated the transdifferentiation of M1 macrophages to D11b^+^, Gr-1^+^ myeloid-derived suppressor cells (MDSCs) in visceral adipose tissue and the peritoneal cavity of lean and obese mice. This transdifferentiation has an anti-inflammatory outcome.	[29]
Brazil	Ethanolic extract	8.0% artepillin C and 0.14% culifolin	Propolis, especially artepillin C, increased the TNFR2 expression through the IRF4/cMyc axis in Tregs.	[30]
China	Adjuvant in experimental vaccine	Not determined	The parameters evaluated were relative percent survival (RPS), the specific IgM antibody level and the expression profiles of several immune-related genes. The results showed propolis could effectively protect the experimental fish against *A. salmonicida* infection with an RPS of 89.47%. The gene expression data revealed induction of differential expression of the pro-inflammatory cytokines, MHC class I, T-cell markers and Ig markers in immunized fish, with higher levels in liver and spleen than in head kidney and gill.	[31]
Egypt	Hydroethanolic extract	Galangin, pinostrobin, chrysin, pinobanksin-3-acetate, luteolin, pinocembrin, formononetin, pinobanksin, apigenin, biochanin A, quercetin-3-methylether, dimethylallylcaffeate, phenylethylcaffeate, quercetin-3,3′-dimethylether, quercetin-7-methylether	In newborn Egyptian-Nubian goat kids, propolis supplementation significantly increased the serum IgG and IgA immunoglobulin levels and reduced the serum cytokine levels (IFN-γ, TNFα, IL1β, and IL6).	[32]
Indonesia (*Trigona sp*.)	Ethanolic extract	Phenolics, 3-methoxy- (CAS) m-guaiacol (phenol) and phenyl ester (CAS) phenyl carbamate (carbamic acid) guaiacol	In LPS-treated zebrafish, propolis lowered the expression of *C3*, *C1r/s*, C6, and *Bf* genes. Propolis also inhibited the migration of macrophages in the LPS-treated zebrafish.	[33]
Iran	Adjuvant of aqueous and Alcoholic extract	Not determined	In HIV-1 polytope vaccine candidate, propolis induced lymphocyte proliferation, IFN-γ and IL-4, and IgG responses.	[34]
Taiwan	Ethanolic extract	Not determined	By giving propolis orally in mouse, the increase in neutrophil infiltration caused by intraperitoneal injection of MSU was greatly decreased. Propolis also inhibited MSU-induced IL-1β, active caspase-1, IL-6, and MCP-1 expression in peritoneal lavage fluid.	[26]
Not determined	Propolis powder	8% Artepillin C	In allergic inflammation mouse model, propolis suppressed the IgE/antigen-induced expression of IL-4, IL-6, and IL-13 in basophils. Phosphorylation of FcεRI signaling molecules Lyn, Akt and ERK was downregulated in basophils treated with propolis. Propolis also inhibited IgE-CAI and attenuated intestinal anaphylaxis, which involves basophils and basophil-derived IL-4.	[35]

**Table 2:** Anti-inflammatory properties of propolis in cancers.

Geographical Sources of Propolis	Types of Extract	Bioactive Compounds	Measured Outcome	References
**In vitro and ex vivo**				
China	Ethanolic extract and CAPE	CAPE	Propolis and CAPE reduced the expression of TLR4 signaling pathway molecules such as TLR4, MyD88, IRAK4, TRIF and in MDA-MB-231 breast cancer cell line.	[39]
China	Ethanolic extract	Caffeic acid, *p*-coumaric acid, ferulic acid, isoferulic acid, cinnamic acid, kaempferol, quercetin, 3,4-dimethoxycinnamic acid, apigenin, pinobanksin, chrysin, pinocembrin, galangin, CAPE, 3-*O*-acetyl pinobanksin	In human melanoma cell A375, propolis downregulated the expression of pro-inflammatory protein NLRP1 (NLR family pyrin domain containing 1) and the mRNA of pro-inflammatory cytokines IL-1α, IL-β and IL-18.	[44]
China	Ethanolic extract	Caffeic acid, *p*-coumaric acid, ferulic acid, resveratrol, quercetin, apigenin, kaempferol, chrysin, pinocembrin, galangin, CAPE	In MDA-MB-231 cells, propolis reduced the expression of proinflammatory cytokines, including tumor necrosis factor-alpha (TNF-*α*), interleukin (IL)-1*β*, and IL-6, and NLRP3 inflammasome.	[47]
**In vivo**				
Brazil	Ethanolic and water extracts	Not determined	Animal models of colorectal cancer. Only ethanolic extract reduced the tumor volume and the multiplicity of colorectal carcinomas. Ethanolic extract of propolis reduced the expression of inflammation-associated proteins: inducible nitric oxide synthase (iNOS), TNF-α, NF-κB and glutathione peroxidase-2.	[45]
Not determined	Hydroethanolic extract	Not determined	Breast cancer model in rats. The animals were fed 50, 100, and 200 mg propolis extract/kg body weight of propolis daily for 4 weeks. Propolis treatments reduced the population of IL-10 and TGF-β Expressing-CD4+CD25+ Regulatory T cells.	[41]
**Human clinical trials**				
Iran	Not mentioned	Not determined	Significant decrease in the antioxidant-oxidant balance in the Propolis group. There was increased serum level of the anti-inflammatory factor TGF-β. The serum levels of MMPs were decreased in the Propolis group.	[46]

**Table 3:** Anti-inflammatory properties of propolis indental/ oral-related disorders and diseases.

Geographical Sources of Propolis	Types of Extract	Bioactive Compounds	Measured Outcome	References
**In vitro and ex vivo**				
Bulgaria	Hydroalcoholic extract of propolis	Caffeic acid, Pinocembrin, 1,3,8-Trihydroxy-6-methylanthraquinone, Chrysin, 3,4,5-Trihydroxybenzoic acid ethyl ester, α-Linolenic acid, Apigenin, Galangin, Linoleic acid, Stigmasterol, Ferulic acid, β-sitosterol, p-Coumaric acid, 3-Hydroxy-4-methoxy-Cinnamic acid iso-Ferulic acid, 3,4-Dimethoxycinnamic acid, Benzoic acid, Caffeic acid phenetyl ester, hydroquinone, vanillin, trans-Cinnamic acid, Protocatechuic acid, 2,5-Dihydroxyacetophenone, Hydrocinnamic acid, p-Hydroxybenzoic acid, m-Hydroxybenzoic acid	Ex vivo: gingival crevicular fluid (GCF) in adolescents with moderate gingivitis Cytokine production (IL-1β, IL-6, TNF-α, IL-17A, IL-18, and INF-γ) was significantly reduced in GCF of adolescents with gingivitis 20th day after receiving toothpaste containing propolis.	[50]
Brazil	Dissolved in DMSO	Not determined	In human periodontal ligament cells, propolis reduced the genes and protein expression levels of IL-1β, IL6, and IL8.	[51]
Brazil	Ethanolic extract	Not determined	hDPC viability was slightly higher in 10 μg/mL of EEP compared to MTA group, with a significant increase in cell numbers. A significantly higher level of Alizarin Red staining was exhibited in the EEP group. The expression levels of the odontogenic marker genes ALP, DSPP-1, and OCN were significantly increased in the EEP group. A significant decrease in IL-1β and IL-6 mRNA expression was observed in the EEP group. Brazilian propolis exhibited similar cell viability to that of MTA. It had significantly higher anti-inflammatory and mineralizing effects on hDPCs than did MTA.	[52]
Indonesia (*Trigona* sp.)	Ethanolic Extract Propolis, Flavonoid-Propolis, Extract Non-Flavonoid	Not determined	Ethanolic extract was the most effective in reducing the expression of IL-6 in inflamed dental pulp tissue.	[53]
Taiwan	Ethanolic extract	Propolin C, D, F, G, and H	In human gingival fibroblasts, propolis inhibited Taiwanese green propolis (TGP) inhibits high glucose-induced NLRP3 inflammasome activation, evidenced by the downregulation of NLRP3, caspase-1 and IL-1β mRNA and protein levels.	[54]
**In vivo**				
Indonesia	Ethanolic extract incorporate into gel	Not determined	Propolis increased the expression of TGF-β in the tension side of alveolar bone in the orthodontic tooth movement animal models.	[57]
Iraq	Ethanolic extract	Not determined	Irrigation with propolis sample as an adjunct to SRP, significantly decreased the serum IL-1β, TNF-α, and MDA levels in experimental periodontitis rats when compared with rats that were treated by SRP with vehicle irrigation.	[58]
Not determined	Ethanolic extract	8.0% artepillin C and 0.14% culifolin	In gingival irritation model mice, topical application of propolis ointment promoted wound healing and reduced the infiltration of inflammatory the expression of IL-1β and TNF-α. In human gingival fibroblasts, propolis suppressed an increase in IL-1β and TNF-α upon stimulation with H_2_O_2_	[55]
Not determined	Not determined	Not determined	In irradiated rats with tongue damage and oral mucositis, propolis reduced the expression of myeloperoxidase and TNF-α.	[56]
**Human clinical trials**				
Brazil	Aqueous extract	Not determined	Patients subjected to gingival curettage treated with propolis had better improvement in terms of plaque index (PI), probing pocket depth (PPD), bleeding on probing (BOP), and reduction in interleukin-1β (IL-1β), compared to patients that were prescribed 1% tetracycline.	[59]
China	95% of Propolis powder (poplar type) mixed with 0.3 mL of normal saline (1:1.5 wt./vol) to form a paste	Quercetin, galanga, and chrysin (indentified by the manufacturer)	Only at 4 h did the patients in the propolis group have lesser pain scoresthan the calcium hydroxide group. Acute increase in pain scores (flare-up) was slightly higher in the propolis group.	[60]
Not determined	Ethanolic extract—topical gel	Not determined	Topical propolis gel application was as effective as 0.1% triamcinolone acetonide in reducing pain and erythema in symptomatic oral lichen planus.	[61]
Not determined	Propolis powder in 70% ethyl alcohol	Not determined	In primary teeth from children aged 5-10 years following pulpotomy, propolis treatment appeared to be more efficacious compared to standard treatment Formocresol based on histological examination showing thick and continuous dentin bridge formation with minimal inflammation (leukocyte infiltration).	[62]
Not determined	Not determined	Not determined	Propolis-containing toothpaste significantly reduced the plaque accumulation and inflammatory response, especially the IL-1β and IL-6 levels in patients with gingivitis.	[63]
Not determined	Hydroethanolic extract	Not determined	In patients with leukemia treated with chemotherapy, patients in the propolis treatment had lower incidence of oral mucositis compared to the control group that were given traditional Chinese medicine. The propolis group patients had significantly shorter oral mucositis recovery time. The mRNA expression of inflammatory cytokines such as IL-22 and TNF-α, and chemokines CXCL9 and CXCL10 were also significantly less in the propolis group.	[64]
Not determined	Not determined	Not determined	Propolis mouthwash significantly reduced papillary bleeding index.	[65]

**Table 4:** Anti-inflammatory properties of propolis inmetabolic syndrome-related disorders and diseases.

Geographical Sources of Propolis	Types of Extract	Bioactive Compounds	Measured Outcome	References
**In vitro**				
China	ethyl acetate extract	Caffeic acid, *p*-coumaric acid, ferulic acid, isoferulic acid, CAPE, apigenin, chrysin, quercetin, kaempferol, galangin, pinocembrin, pinobanksin	Propolis ameliorated ox-LDL induced human umbilical vein endothelial cells (HUVECs) injury by inhibiting LOX-1 level and ROS production. Propolis activated PI3K/Akt/mTOR pathway and deactivated p38 MAPK to inhibit apoptosis and autophagy in modulating inflammation.	[75]
**In vivo**				
Brazil	Not determined	Prenylated derivatives of cinnamic acid including artepillin C (6.1%)	Infiltration of epididymal fat by macrophages and T cells was reduced in the propolis group in mice fed with high fat diet.	[71]
Brazil	Hydroethanolic extract	Poplar: caffeic acid, coumaric acid, ferulic acid, apigenin, CAPE, quercetin, kaempferol, galangin, cinnamic acid, pinocembrin, chrysin Baccharis: caffeic acid, ferulic acid, quercetin, kaempferol, pinocembrin, artepillin C Dalbergia: formononetin, biochanin A, liquiritigenin, vestitol, medicarpin	In high-fat-fed mice, poplar propolis reduced the expression of mRNAs coding for inflammation, namely TNF-α and chemokine C-C motif ligand 5 (Ccl5) and Ccl2. However, Baccharis and Dalbergia propolis appeared to increasemRNA expression.	[76]
China	Ethanolic extract	Protocatechuic acid, caffeic acid, p-coumaric acid, ferulic acid, daidzein, cinnamic acid, quercetin, naringenin, genistein, keampferol, hesperetin, and biochanin A	EEP treatment reduced insulin resistance induced by high fat diet (HFD)-fed. EEP supplementation improved impaired glucose tolerance and reduced the gain in body weight and fat accumulation in HFD-fed mice. EEP treatment significantly reduced the mRNA expression levels of TNF-α, IL-1β, and IL-6 in a dose-dependent manner, but increased that of IL-10 in hepatic and adipose tissues.	[72]
China	Ethanolic extract	3-*O*-acetyl pinobanksin, chrysin, pinocembrin, pinobanksin, and CAPE	In hypercholesterolemia rabbits, propolis ameliorated restenosis by reducing the plasma levels of C-reactive protein, interleukin-6, and tumor necrosis factor-α (TNF-α), and inhibiting the expression of CD68, TLR4, NF-κB p65, MMP-9, and TNF-α in the carotid arteries	[73]
France	Ethanolic extract	Caffeic acid, coumaric acid, ferulic acid, apigenin, CAPE, quercetin, kaempferol, galangin, cinnamic acid, pinocembrin, chrysin	PPEE supplementation reduces the HF-mediated adiposity index, adipocyte hypertrophy, and body weight gain. It also improves HOMA-IR and fasting glucose levels. Gene expression profiling of adipose tissue (AT) shows an induction of mRNA related to lipid catabolism and mitochondrial biogenesis and inhibition of mRNA coding for inflammatory markers. Interestingly, several Nrf2-target genes are induced in AT following administration of PPEE. The ability of PPEE to induce the expression of Nrf2-target genes is studied in adipocytes. PPEE is found to transactivate the Nrf2 response element and the Nrf2 DNA-binding, suggesting that part of the effect of PPEE can be mediated by Nrf2.	[74]
Indonesia	Ethanolic extract	Not determined	Diabetic mice. The animals were fed 50, 100, and 200 mg propolis extract/kg body weight of propolis daily for 2 weeks. Propolis treatments reduced the expression of CD4+IFN-γ+ and CD4+TNF-α+ T cells in diabetic mice to the level observed in normal mice.	[77]
Indonesia	Hydroethanolic extract	Not determined	Propolis treatment reduced the expression of pro-inflammatory markers such as NF-κB, TNF-α, and interferon-γ on macrophages and TLR3 and TLR4 on B cells, isolated from diabetic mice.	[78,79]
Malaysia (*Heterotrigonaitama*)	Hydroethanolic extract	Not determined	In streptozotocin-induced diabetic rats, propolis attenuated the increase in the expression of nuclear factor kappa B (NF-κB), tumor necrosis factor-α, interleukin(IL)-1β and caspase-3. In addition, propolis increased the expression of IL-10 and inhibited the proliferation cell nuclear antigen in the liver of diabetic rats. Propolis also ameliorated the inflammation of the liver by reducing the number of the hepatocytes with pyknotic nuclei and inflammatory infiltration.	[80]
Taiwan	Ethanolic extract	Propolins C, D, F, G, and H	In Streptozotocin/High-Fat Diet-induced rats, propolis delayed the progression of type 2 diabetes mellitus by reducing the severity of β-cell damage indicated by the HOMA- β and β-cell mass. Propolis also significantly reduced the pro-inflammatory cytokines especially TNF-α and IL-1β.	[81]
Turkey	Ethanolic extract	1-Hexen-4-yne,3-ethylidene-2-methyl; 1,4-Bis[(3-methyl-5-oxo-1-phenyl-2-pyralozin-4-ylidene)methyl]benzen; N,N’,N”,N”-Tetraphenyl (2-imidazolidenyl) imidazole; Benzene, 1,3-bis(3-phenoxyphenoxy); 1H-NAPHTHO [2,3-C] pyran-3-acetic acid 5-[[(1,1-dimethylethyl) aimethylsilyl] oxy] 3,4-dihydro-10-hydroxy-9-methoxymethyl-,methyl ester, cis-(+-)-; 2-Methoxyethoxy-2-methylethyl-(2-hydroxy-1-methyl)ethoxy]-1-methylethyl ether; .delta.-Terpineol; 4-Methyl-1,4-heptadiene; Ethanol, 2-(2,3-butadienyloxy)-ethene, ethyloxy-; Ethanol,2-(2-chloroethoxy); Ethanol,2-(1-methylethoxy); Tetracyclo [3.3.00(2,4).0(3,6)]oct-7-ene; 1-Phenyl-3-(2-nitrophenyl)-2-pyrazoline; 1,2-Pentadiene; 1,3,7-Octatrien-5-yne; 4-Methylbenzenamide, N-benzylidenamino-; Benzene, ethynyl-; Benzene, 1-methyl-4-nitroso-; 1-Phenyl-1,2-bis(phthalimidooxy)ethane; Mercury, chlorophenyl-; Acetaldehyde, chlorodifluoro-	Propolis reduced the serum level of IL-6 and TNF-α in non-alcoholic fatty liver disease animal model.	[82]
Turkey	Ethanol extract of propolis	Not determined	When compared to the L-NAME group, NOS-inhibited (hypertensive) rats treated with propolis had significantly lower levels of NF-κB in testis tissue.	[83]
Turkey	Hydroethanolic extract	Not determined	In cardiovascular rat models, propolis reduced the expression of d NF-κB levels.	[84]
**Human clinical trials**				
Brazil	Ethanolic extract	Caffeic acid, *p*-coumaric, 3,5-dicaffeoyl quinic acid, 4,5-dicaffeoyl quinic acid, aromadendrin, drupanin, artepellin C, bacharin	The marker of inflammation MCP-1 (monocyte chemoattractant protein-1) level in urine gradually decreases in the propolis-treated CKD patients’ group	[85]
Brazil	Ethanolic extract	Minimum of 11 mg of artepillin C	In a 24-month trial, propolis-treated elderly subjects had significant improvement in MMSE scores compared to placebo group. The cognitive improvement correlated with the decrease in serum pro-inflammatory cytokines IL-1β, IL-6, and TNF-α. Propolis-treated group also had an increase in serum TGF-β1.	[86]
China	Ethanolic extract of propolis	Not determined	In diabetic patients, propolis increased serum GSH, flavonoids, and polyphenols. In addition, propolis decreased serum lactate dehydrogenase. In addition, serum IL-6 was significantly increased in the Chinese propolis group.	[87]
Iran	Not determined	Total flavones and flavonols: 8.4%, total flavanones and dihydroflavonols: 4.6% and total phenolic compounds: 28%	Patients with Type 2 Diabetes Mellitus. In comparison to the placebo group, blood levels of high-sensitivity CRP and TNF-α significantly reduced after the administration of Iranian propolis. On day 90, there was a decrease in serum IL1-β and TNF-α in the propolis group.	[88]
Iran	Ethanolic extract	Not determined	Propolis attenuated hepatic steatosis and fibrosis in patients with nonalcoholic fatty liver disease (NAFLD). Propolis significantly reduced the inflammatory marker hs-CRP.	[89]

**Table 5:** Anti-inflammatory properties of propolis inorgan toxicity and inflammation.

Geographical Sources of Propolis	Types of Extract	Bioactive Compounds	Measured Outcome	References
**In vitro and ex vivo**				
Algeria	Hydroethanolic extract	Kaempferol-methyl ether, galangin, kaempferol, apigenin, CAPE, quercetin, pinocembrin, eriodictyol, chrysin, kaempferol-dimethyl ether, 3,4-dimethyl caffeic acid, kaempferol-methyl ether, caffeic acid benzyl ester, myricetin-3,7,4′,5′-tetramethyl ether, *p*-coumaric acid, *p*-coumaric acid methyl ester, caffeic acid cinnamyl ester, chrysin-5-methyl ether, pinobanksin-3-*O*-butyrate, chrysin-5-methyl ether, pinobanksin-3-*O*-propionate, kaempferide, vitexin	In peripheral blood mononuclear cells of Celiac disease patients, propolis reduced the expression of nitric oxide and interferon-γ and significantly increased IL-10 expression. Propolis also downregulated the iNOS expression and the activity of NFκB and pSTAT-3 transcription factors.	[92]
Chile	Ethanolic extract of propolis (EEP)	Caffeic acid, *p*-Coumaric acid, Isoferulic acid, Ferulic acid, Dihydrokaempferol, Benzoic acid, 3,4-Dimethyl-caffeic acid, 3,4-Dimethyl-caffeic acid isomer, Pinobanksin-5-methyl ether *p*-coumaric acid methyl ester, Quercetin, Pinobanksin, Luteolin, Quercetin-3-methyl ether, Kaempferol, Pinocembrin-5-methyl ether, Apigenin, Isorhamnetin, Kaempferol-methyl ether, Chrysin-5-methyl ether, Cinnamylidenacetic acid, Pinobanksin acetate derivative, Rhamnetin, Pinocembrin, Caffeic acid benzyl ester, Caffeic acid isoprenyl ester, Pinobanksin-3-*O*-acetate, Chrysin, Caffeic acid phenylethyl ester (CAPE), Galangin, Acacetin.	Nitric oxide (NO) levels in rabbit chondrocytes were significantly increased following IL-1β stimulation, whereas they were significantly decreased following EEP 2.5 µg/mL therapy.	[98]
China	Ethanolic extract of Chinese propolis (EECP)	Cafeic acid, P-Coumaric acid, Ferulic acid, Isoferulic acid, Cape, Apigenin, Chrysin, Quercetin, Kaempferol, Galangin, Pinocembrin, Pinobanksin	In LPS-stimulated Human umbilical vein endothelial cells (HUVECs), EECP administration significantly prevented NF-κB p65 from translocating from the cytoplasm to the nucleus and significantly reduced TLR4 expression.	[99]
**In vivo**				
Algeria	Hydroethanolic extract	Not determined	Propolis reduced the expression of NO and TNF-α levels in the α-Tropomyosin-induced retinal damage. Propolis also down-regulated NOS2 and NF-κB expression in the retina. In addition, propolis also attenuated the retinal damage by inducing Th2 response.	[100]
Algeria	Ethyl acetate extract	Flavonoids: catechin, quercetin, rutin, acacetin, chlorogenic acid, apigenin, pinocembrin, chrysin, kaempferol, thymol; phenolic acids: ferulic acid, gallic acid, caffeic acid, ellagic acid, m-coumaric, rosmarinic acid, trans-cinnamic; ascorbic acid; benzoic acid; galangin; tectochrysin	PGE2 production measured from peritoneal exudates and TNF-α levels in the liver are reduced by the administration of EAP at doses of 100 and 250 mg/kg in epirubicin-induced hepatotoxicity rats.	[101]
Algeria	Ethanolic extract	Catechin, quercetin, rutin, acacetin, chlorogenic, apigenin, pinocembrin, chrysin, kaempferol, Thymol, ferulic acid, gallic acid, caffeic acid, ellagic acid, rosmarinic acid and trans-cinnamic acid; m-coumaric and ascorbic acid	In epirubicin-induced cardiotoxicity and nephrotoxicity rat models, propolis attenuated necrosis, edema, and inflammatory infiltrates in the heart and kidney cells.	[102]
Algeria	Ethyl acetate phase of ethanolic extract	Not determined	Propolis reduced the expression of TNF-α and prostaglandin E2 in associated with the inflammation- paw edema. Propolis also reduced the infiltration of white blood cells (neutrophils, eosinophils, basophils, lymphocytes) in the edema.	[103]
Australia	Dry extract	Caffeic acid, ferulic acid, sinapic acid, rosmarinic acid, cinnamic acid, quercetin, apigenin, kaempferol	In doxorubicin-induced multi-organ toxicity animal models, propolis reduced the expression of IL-1β.	[104]
Brazil	Hydroalcoholic	Liquiritigenin, formononetin, biochanin A, and daidzein	Ulceritis colitis animal model, treated with 10 (P10) and 100 (P100) mg/kg/day for 7 days. The histological assessment showed the protective effect of propolis compared to control. P10 treated animals had lymphocyte-rich inflammatory infiltration, whereas polymorphonuclear-rich infiltration shown in the P100. P10 had lower inflammatory scores and histological damage compared to P100 and control. Propolis downregulated myeloperoxidase (MPO) activity.	[93]
Brazil	Not determined	Not determined	Mice were exposed to chronic cigarette smokes for 60 days to develop a condition mimicking the chronic obstructive pulmonary disease (COPD) and treated with propolis orally for another 60 days. Histological analysis showed that propolis recovered alveolar spaces, alveolar septa, and elastic fibers. Propolis also increased and decreased the expression of MMP-2 and MMP-12, respectively—promoting the process of tissue repair. Propolis recruited leukocytes, including macrophages, without ROS release. Propolis appeared to promote lung repair via via macrophage polarization from M1 to M2 and the downregulation of IGF1 expression in a Nrf2-independent manner.	[105]
Brazil China	Ethanolic extract	Protocatechuic acid, vanillic acid, caffeic acid, *p*-coumaric acid, ferulic acid, trans-isoferulic acid, 3,4-dimethoxycinnamic acid, gallic acid, cinnamic acid, quercetin, pinobanksin, luteolin, hesperitin, kaempferol, galangin, pinocembrin, 3-O-acetylpinobanksin, chrysin, CAPE, apigenin, artepillin C	Propolis reduced the inflammatory markers in the colonic inflammation: IL-1β, IL-6 and MCP-1. Propolis also increased the expression of TGF-β. Further, propolis increased the diversity and richness of gut microbiota populations. Both forms of propolis reduced the populations of *Bacteroides* spp.	[95]
Brazil	Ethanolic extract	Not determined	Propolis reduced the severity of acetaminophen-induced hepatocellular necrosis by reducing inflammatory cell infiltration and downregulating mRNA expression of IL-10 and IL-1β.	[106]
Brazil	Hydroethanolic extract	Daidzein, formononetin, and biochanin A	Topical application of propolis was as effective as a commercially available chemical sunscreen oxybenzone in ameliorating inflammation caused by UVB exposure by reducing mononuclear inflammatory infiltrate.	[107]
Brazil	Ethanolic extract	Not determined	L-arginine was administered in groups two and three to cause Acute Pancreatitis. Two 250 mg/100 g of body weight (BW) intraperitoneal (IP) injections of L-arginine produced in isotonic saline (20 percent 0.15 M sodium chloride) were given one hour apart. After two hours after L-arginine injection, the rats in group three received daily treatments of Brazilian green propolis alcohol extract at a dose of 100 mg/kg BW for seven days. Serum analysis of proinflammatory (IL-1β, IL-6, and TNF-α) was lower in group three (propolis-treated acute pancreatitis rat group) than in group two (acute pancreatitis rat group). Propolis increase the production of the anti-inflammatory (IL-22) cytokine in serum of acute pancreatitis rat group	[108]
Brazil	Ethanolic extract	Not determined	Propolis inhibits the mRNA expression of IL-1β and IL-6 from methylglyoxal (MGO) treatment in extensor digitorum longus (EDL) muscle	[109]
Brazil	Green propolis dissolved in PBS	Not determined	Expression of HIF-1α and GFAP was increased, and the level of histone acetylation decreased in saline-treated ischemic retinas within 7 days. BGP treatment effectively attenuated the elevated expression of HIF-1α, GFAP, Bax, NF-κB and p53. The expression of Bcl-2, Nrf2, HO-1 and the level of histone acetylation increased by BGP treatment, resulting in a significant difference between BGP-treated and saline-treated retinas. Immunohistochemical staining for Brn3a also revealed that BGP treatment protected against RGC loss in ischemic retina.	[110]
Brazil	Red propolis Ethanolic and hydroalcoholic extract	Not determined	Pre-treatments with the HERP at different doses (50, 250, or 500 mg/kg) inhibited the total lesion areas in a dose dependently fashion.The gastric tissue of the animals treated with HERP (250 and 500 mg/kg, p.o.) showed lesser mucosal damage.The treatment with 50 and 250 mg/kg HERP in the pylorus ligature model of gastric secretion significantly reduced secretion volumes (*p* < 0.01) and increased H^+^ concentrations (50 mg/kg, *p* < 0.01). The production of mucus was significantly increased with HERP (500 mg/kg) treatment. HERP treatments reduced the diameter of the inhibitory zone to 13.0 ± 2.0 mm at 100 mg/mL.	[111]
Brazil	Green propolis—hydroalcoholic extract	Caffeic, coumaric, ferulic and cinnamic acids, aromadendrin-4′-*O*-methyl ether, isosakuranetin, drupanin, Artepillin C, and baccharin	Pre-treatment with HEGP, at doses of 100 and 300 mg/kg was able to reduce the ulcer area by 54% (17.60 ± 3.12 mm^2^) and 96% (1.48 ± 0.27 mm^2^), respectively. HEGP (100 and 300 mg/kg) showed reduction in histological changes inthe gastric mucosa. HEGP (100 and 300 mg/kg) showed reduction in histological changes in the gastric mucosa. Treatment with HEGP accelerated the healing process promoting a notable recovery of the ulcer base. The pre-treatment with HEGP (300 mg/kg, p.o) increased the mucin-like glycoproteins staining by 67%. HEGP treatment showed ducts of glands with less damage, the ducts with higher amounts of mucus and a basal region with intense PCNA staining, suggesting the enhancement in cellular proliferation. HEGP had a direct DPPH scavenging radical ability in a concentration-dependent manner. Oral pre-treatment with HEGP (300 mg/kg) reduced the ROS production as well as lipoperoxidation levels.HEGP at doses of 100 or 300 mg/kg reduced the SOD activity by 11% and 26%, and increased the CAT activityby 77% and 82%, respectively. Moreover, the GST activity was increased by 20% in animals that received HEGP at 100 or 300 mg/kg. HEGP prevented the increase in MPO activity in ethanol-induced acute gastric ulcer, reducing the activity by 93%.	[96]
Brazil	Hydroethanolic extract	Phenolics and flavonoids	In rats exposed to excessive UVB irradiation, propolis was shown a photoprotective properties: UV-B exposed rats had comparable appearance to control rats where mast cell infiltration was comparable to controls.	[112]
Brazil	Ethanolic extract	CAPE, *p*-coumaric acid, trans-cinnamic acid, aromadendrin, artepillin C	In septic rat model, propolis attenuated the increase in the expression of Toll-like receptor 4 and nuclear factor-kappa B proteins in lung and renal tissues and macrophage infiltration. Propolis also inhibited the increase in cytokines IL-17a, IL-12p70, IL-1β, TNF-α	[113]
Brazil	1% gum arabic extract	Not determined	Propolis significantly reduced inflammation in the cyclophosphamide-induced hemorrhagic cystitis in rats.	[114]
China	Ethanolic extract	Galangin, apigenin, *p*-coumaric acid, kaempferol, quercetin, chrysin, caffeic acid phenethyl ester, and artepillin C	In nicotine-induced pulmonary and hepatic damage, propolis significantly reduced Nrf2 expression and increased HO-1 expression in a dose-dependent manner. Propolis also reduced the expression of iNOS and TNF-α.	[115]
China	Hydroethanolic extract		In mice with alcohol-induced depressive symptoms, propolis reduced the expression of serum levels of LPS and fatty-acid-binding protein 2 (FABP2). Propolis also reduced the inflammatory cytokines: TNF-α, IL-6, and IL-18 in the spleen of the mice.	[116]
Denmark	Aqueous extract of propolis (PWE)	Caffeic acid, ferulic acid, isoferulic acid, vanillin, and cinnamic acid)	PWE was successful in protecting against the elevation in the intestinal marker of inflammation TNF-α and MPO activity in radiation-induced intestinal mucosal injury.	[117]
Egypt	Methanolic extract of propolis	Not determined	In ulcerative colitis, propolis methanol extract administration resulted in a dose-dependently significant reduction in both inflammatory mediators, TNF-α and NO The inflammatory enzymes COX-1 and COX-2 could be inhibited by propolis methanol extractin vivo.	[94]
Egypt	Ethanolic extract of propolis dissolved in 0.5% carboxyl methyl cellulose (CMC)	Not determined	Propolis pretreatment of endotoxemic rats was effective in controlling the depletion of renal GSH content and its correlated enzymes. Despite the renal inflammatory marker IL-1β, PGE2, NO contents, Bax\Bcl 2 ratio, and NF-kB activation were greatly reduced by propolis and comparator group.	[118]
Egypt	Hydroethanolic extract	Not determined	In mice with CCl_4_-induced chronic liver fibrosis, propolis attenuated the toxic effect of CCl_4_ through several mechanisms such as: 1. Propolis supplementation attenuated the altered distribution of CCl_4_-mediated T and B cells in spleen and lymph node tissues. 2. Propolis restored the levels of TGF-β, proinflammatory cytokines, ROS, and NO increased by CCl_4_ in lymphoid tissues.	[119]
Egypt	Hydroethanolic extract in the form of nanoparticles	Cholesta-9(11),20(22)-dien-23-one, 3,6-dih; N-p-toluenesulfony-tyrosyl-S-carboxymethyl; Benzotriazole, 1-(tetrafluoropyrid-4-yl)-; 2,1,3-Benzoxadiazole-5-carboxylic acid, 3-ox; Propionic acid, 3-tetrazol-1-yl; Propionic acid, 3-tetrazol-1-yl; Pyrimidine-4,6(3H,5H)-dione, 2-butylthio; Thiamphenicol; 1,3,5-Triazine-2,4-diamine, N,N0 -bis(1-me; n-Propylamine, N-acetyl-3-[2-acetyl-3,4,5; 4H-Cyclopenta[b]thiophene-2,5-dicarboxy; Bis [1,3-bis(diisopropylphosphino)propane-c; 3-Acetyl-2-(2,4-dimethoxy-phenylamino)-6 methyl; 4-tert-butyl-2,6,7-trioxa-1-phosphabicyclo[;1,2,5-Oxadiazole-3-acetic acid, 4-methyl; Dimethylacetylmethyl phosphonate; Androstan-17-ol, 5,6-epoxy-3-fluoro-, aceta; Naphto [3,2,1-c,d]isoindol-4(5H)-one, 2-hy; 1,6-Dideoxy-2,4-monoethylene-d-altritol; 1-Propanol, 2-methyl; Phenylethyl alcohol; 2-Propen-1-ol, 3-phenyl; Phosphonic acid, [3-(acetylamino)propyl]-; 2-Propen-1-ol, 3-phenyl; n-Hexadecanoic acid; 9,10-Secocholesta-5,7,10(19)-triene-3,25; Benzene, 1-(1,5-dimethyl-4-hexenyl)-4-methyl; Erucic acid; 5-Azulenemethanol, 1,2,3,4,5,6,7,8-octahydr; Bicyclo [2.2.1]heptane-2-thione, 1,7,7-trimethyl; Acetamide, N-[3-hydroxy-3-methyl-4-[(4-nit; Ferrocene, 1,2,3,4-tetrachloro-; Cyclohexanemethanol, 4-ethenyl.alpha.,.alpha.,4-; Ergostane-3,5,6,12,25-pentol, 25-acetate, (3.be; 2-Brom-uridine; Pentadecanoic acid; 5-Heptenoic acid, ethyl ester, (E)-; Ferrocene, 1,2,3,4-tetrachloro-	Propolis attenuated the CCl_4_- induced damage in kidney and liver by inhibiting the increase in transforming growth factor β (TGF-β)—liver, nephrin—kidney, and caspase-9 and the decrease in Bcl-2 levels.	[120]
India	Hydroethanolic and aqueous extracts	Not determined	Both extracts at a dose of 400 mg/kgBW were effective at reducing the expression of NF-κB and COX-2 in liver tissue of Rifampicin and Isoniazid treated groups.	[121]
Indonesia (*Trigona* sp.)	Ethanolic extract of propolis (EEP)	Not determined	Treatment with EEP at a dose of 30 mg/200 gBW/day resulted in a notable reduction in protease activity and the least amount of damage to the trachea’s histological structure in rats exposed to cigarette smoke.	[122]
Indonesia	Ethanolic extract of propolis	Not determined	Compared to traumatic brain damage without therapy, propolis administration greatly enhanced Hsp70 expression and significantly decreased apoptotic markers (caspase 3, AIF, and TUNEL test).	[123]
Indonesia (*Tetragonula sapiens*)	Propolis powder	Phenolics and flavonoids	Propolis reduced the carrageenan-induced edema in rats.	[124]
Japan	suspended in 1% gum acacia in water	Not determined	Propolis reduced the severity of anti-TB drugs-induced hepatic injury. Histopathological and electron microscopic observations demonstrated the hepatoprotective potential of propolis by reducing the expression of TNF-α and IL-6 and increasing the IGF-1expression.	[125]
Korea	Ethanolic extract	Not determined	In phorbol-12-myristate-13-acetate-induced ear edema model, the expression of iNOS and COX-2 generated by LPS stimulation was reduced by the propolis in a dose-dependent manner	[22]
Mexico	Hydroethanolic extract	Catechol, catechin, chrysin, naringin, naringenin, pinocembrin	In the indomethacin- induced gastric ulcer animal models, propolis exhibited gastroprotective properties by increasing PGE_2_ level and reducing the expression of inflammatory cytokines such as TNF-α, IL-1β, and IL-6.	[97]
New Zealand	Not determined (complexed with gamma- cyclodextrin)	Caffeic acid, *p*-coumaric acid, cinnamic acid, cinnamic acid, caffeate esters, 3-methyl-3-butenyl caffeate, CAPE, pinobanksin, pinocembrin, pinobanksin-3-acetate, chrysin, galangin, pinostrobin, tectochrysin, isalpinin	In rats exposed to urban dust, propolis supplementation reduced lung TNF-α, IL-4, and IL-6 cytokine production. Propolis did not affect immune cell infiltration and lung function.	[126]
Philippines (*Tetragonulabiroi* Friese)	Hydroethanolic extract	Pinobanksin-5, 7-dimethyl ether, artepillin C, apigenin, quercetin, luteolin-5-methyl ether, pinobanskin 3-O butyrate or isobutyrate, and kaempferol	Propolis attenuated carrageenan-induced hind paw edema and plasma TNF-α level.	[127]
Turkey	Water-soluble propolis extract	Not determined	Propolis pretreatment significantly reduced I/R-induced IL-6 level elevation in the ovary. Caspase-3 and TNF-α immunoreactivity in the ovary was minimal in the I/R (ischemia-reperfusion injury) + Propolis group.	[128]
Not determined	Not determined	Not determined	Propolis alleviated the severity of concanavalin A-induced hepatitis. Propolis attenuated the increase in serum level of the inflammatory cytokines tumor necrosis factor-α (TNF-α) and interleukin-6 (IL-6) as well as the profibrotic cytokine TGF-β.	[129]
Not determined	Not determined	Not determined	Propolis alleviated the inflammation in nitrosamine-induced liver injury in animals.	[130]
Not determined	Ethanolic extract	Not determined	When compared to control mice, the CCl_4_-treated mice group showed a noticeably higher expression of HSP70. propolis caused a significant decrease in the expression level of HSP70 when given orally to CCl_4_-treated mice. When compared to the liver lysates from control animals, CCl_4_ significantly increased the amounts of these proinflammatory cytokines (IL-1β, IL-6, and TNF-α). The levels of proinflammatory cytokines in CCl_4_-treatedmice were markedly reduced in mice treated with propolis supplementation.	[131]
Not determined	Not determined	Not determined	When compared to healthy control hamsters, propolis was effective in reducing the neurotoxic effects of propionic acid (PPA), showing minimal changes in IL-6 and IL-10 levels.	[132]
Not determined	Not determined	Not determined	In rats exposed to CCl_4_, propolis attenuated the increase in TNF-α.	[133]
**Human clinical trials**				
Indonesia	Propolis compress	Not determines	The propolis compress treatment group had the highest percentage with a score of 0 or no phlebitis (60%) and the lowest with early signs of phlebitis (40%).	[134]
Iran	Propolis tablet	Not determined	Propolis significantly attenuated the symptoms in moderate persistent asthma patients, compared patients given placebo. Propolis was also shown to reduce eosinophilic inflammation associated with asthma.	[135]
Brazil	Not detetermined	8.0% artepillin C and 0.14% culifolin	In elderly women suffering from rheumatoid arthritis, propolis consumption (24 weeks) did not improve disease severity in terms of Disease Activity Score in 28 joints using erythrocyte sedimentation rate (DAS28-ESR). Propolis also did not affect secondary endpoints, other disease activity assessment (DAS28 using C-reactive protein, simplified disease activity index, clinical disease activity index), ultrasonographic evaluation of synovitis, activities of daily living, quality of life, changes in cytokine levels, and adverse events.	[136]
Brazil	Green propolis	Not determined	Brazilian green propolis demonstrated a tendency to reduce inflammation based on the reduction in serum hs-CRP in the hemodialysis patients.	[137]

**Table 6:** Anti-inflammatory properties of propolis inpathogenic infections.

Geographical Sources of Propolis	Types of Extract	Bioactive Compounds	Measured Outcome	References
Brazil	Alcoholic, glycolic, water-soluble dry extract	Caffeic acid, *p*-Coumaric acid, 3,5-Dicaffeoylquinic acid, 4,5-Dicaffeoylquinic acid, Cinnamic acid, Aromadendrin, Drupanin, Artepillin C, Baccharin	Propolis reduced, in a dose-dependent effect, the viability of *Leishmania (Viannia) braziliensis* promastigotes and helped controlled the parasite burden inside the infected macrophages. Dry propolis extract significantly modified the inflammatory profile of murine macrophages by reducing the TGF-*β* and IL-10 production, while upregulating TNF-*α*. All three types of propolis extract reduced nitric oxide (NO) and superoxide levels in activated *L. braziliensis*-infected macrophages.	[140]
Brazil	Hydroethanolicextract	Not determined	Ex vivo Human-derived peripheral blood mononuclear cells from American Tegumentar Leishmaniasis (ATL) patients and healthy donors, challenged by *Leishmania braziliensis*. Propolis did not reduce the expression of IL-2, TNF-α, IFN-γ and IL-17 in the adherent cells of the healthy donors and ATL patients. When adherent cells from healthy donors were infected with *Leishmania braziliensis*, propolis pre-treatment increased the expression of IL-6, IL-17, and reduced the expression of IL-10.	[141]
Brazil	Ethanolic extract	Caffeoyltartaric acid, 3,4-Dicaffeoylquinic acid, quercetin, 20 gibberellin A7, A9 and A20	Brazilian organic propolis (BOP) has strong anti-inflammatory effects and acts by reducing NF-kB activation, TNF-α release and neutrophil migration. It exhibited antifungal activity on planktonic and biofilm cultures of *Candida albicans*, *C. glabrata*, *C. tropicalis*, *C. krusei* and *C. parapsisolis*, and it reduced in vitro yeast cell adhesion to human keratinocytes at sub-inhibitory concentrations. BOP demonstrated significantly low toxicity in *Galleria melonella* larvae at antifungal doses.	[142]
Iraq	Hydroethanolic extraction followed dissolution in 8% L-lysine	Not determined	Propolis reduced the expression of IL-1β and NLRC4 inflammasome through activation of autophagy following *P. aeruginosa* infection.	[143]
Korea	Ethanolic extract		Korean propolis decreased the levels of these pro-inflammatory cytokines—IL-8, IL-1β, IL-12, and TNF-α, in a dose-dependent manner.It dose-dependently inhibited the expression of COX-2 and iNOS mRNA and protein expression induced by *H. pylori* infection. It also attenuated the phosphorylation of ERK, JNK, and p38 MAPKs in *H. pylori*-infected AGS cells in a dose-dependent manner. It significantly inhibited the NF-κBsignaling pathway in *H. pylori*-infected AGS cells by suppressing IκBα phosphorylation. It not only had radical scavenging activity, which significantly elevated in a dose-dependent manner but also upregulated the protein and mRNA expression of anti-oxidant enzymes, such as HO-1, NQO1, GCLM, and SOD1 dose-dependently. Korean propolis increased the promoter binding activity of Nrf2 to the anti-oxidant relative.	[144]
**In vivo**				
Algeria	Ethanolic extract of propolis (EEP)	Not determined	The concentration of serum TNF-α in the cystic echinococcosis (CE) mice group was significantly reduced after receiving EEP orally, and levels were remarkably similar to those in the Control group. In the spleen of CE mice, there was a significantly elevated iNOS, TNF-α, and NF-κB/p50 immunoreactivity; however, following propolis therapy, this immunoreactivity significantly decreased.	[145]
Egypt	Ethanol Propolis Extract	Not determined	All BHV-1-infected groups had higher blood and cellular levels of TNF-α, IL-2 and IFN-γ after receiving propolis extracts at 50 µg/mL for seven days following inoculation.	[146]
Egypt	Used in powder form as a dietary supplement	Not determined	Dietary PR supplementation was able to (*p* < 0.05) reduce the negative impact of *E. coli* challenge on egg number, egg weight and feed intake by 20%, 7% and 18%, respectively, compared to the laying hens fed on basal diet and challenged with *E. coli*. A significant reduction (*p* < 0.05) was noticed in TNF-α by 13% and in both IL-1β and plasma corticosterone by 37%. Dietary PR minimized (*p* < 0.05) the elevation of MDA concentration induced by *E. coli* challenge by 45% in PR + EC group and retuned back to the normal level compared to control. Data also showed a significant (*p* < 0.05) improvement in the antioxidant status by increasing the concentration of TAC and SOD, by 84% and 20%, respectively, compared to C group. Total white blood cells (TWBCs) were reduced (*p* < 0.05) by 15.8%, while heterophile/lymphocyte (H/L) ratio was increased (*p* < 0.05) by 1.8 folds in PR + EC groups. The lymphocyte proliferation of T-cells was suppressed (*p* < 0.05) by 29.5% and B-cell proliferation was inhibited (*p* < 0.05) by 15.8%, in PR+EC group. PR supplementation caused a significant increase (*p* < 0.05) of villi height. PR significantly (*p* < 0.05) increased call viability (CV)% by 40% compared to C group. PR also normalized the Foxo3 expression to similar levels of the C group	[147]
Saudi Arabia	Hydromethanolic extract	Not determined	*Plasmodium chabaudi* infection model in mice. Propolis reduced oxidative damage by downregulating the malondialdehyde (MDA) and increasing the catalase (CAT) activity and the glutathione (GSH) levels. Propolis appeared to increase the level of pro-inflammatory cytokines such as IFN-γ, TNF-α, GM-CSF and G-CSF.	[148]
Not determined	Ethanolic extract	Not determined	In anthrax animal models, propolis reduced the TNF-α expression.	[149]

## Data Availability

Not applicable.
